# Thonningianin A ameliorated renal interstitial fibrosis in diabetic nephropathy mice by modulating gut microbiota dysbiosis and repressing inflammation

**DOI:** 10.3389/fphar.2024.1389654

**Published:** 2024-08-13

**Authors:** Shujiao Zhang, Shuaixing Zhang, Xuehui Bai, Yaoxian Wang, Yuning Liu, Weijing Liu

**Affiliations:** ^1^ Dongzhimen Hospital, Beijing University of Chinese Medicine, Beijing, China; ^2^ Key Laboratory of Chinese Internal Medicine of Ministry of Education, Beijing Dongzhimen Hospital, Beijing University of Chinese Medicine, Beijing, China; ^3^ Henan University of Chinese Medicine, Zhengzhou, China; ^4^ Renal Research Institution of Beijing University of Chinese Medicine, Beijing University of Chinese Medicine, Beijing, China

**Keywords:** thonningianin A, renal interstitial fibrosis, diabetic nephropathy, gut microbiota, inflammation

## Abstract

**Objectives:**

This study was conducted to examine the potential health benefits of thonningianin A (TA) on renal injury and interstitial fibrosis in diabetic nephropathy (DN) mice.

**Methods:**

In this study, a DN mice model was established using male C57BL/6 mice injected with streptozotocin (STZ, 50 mg/kg) intraperitoneally and treated with TA for 12 weeks. Firstly, the therapeutic and anti-fibrotic effects of TA on DN were evaluated. Secondly, the effect of TA on renal inflammation was evaluated and Western blot was used to detect the changes of NLRP3/ASC/Caspase-1 pathway-related protein expressions in kidney. Furthermore, the effect of TA on impairments in the intestinal mucosa barrier was evaluated and the changes of lipopolysaccharide (LPS) levels in feces and serum were detected by ELISA. Finally, 16S rRNA sequencing was used to detect alteration of gut microbiota diversity and abundance in mice after TA treatment.

**Results:**

The results showed that TA markedly mitigated blood glucose (Glu), decreased 24-h urinary total protein (24hUTP), improved renal dysfunction and kidney index (KI) in DN mice. Furthermore, TA significantly alleviated renal injury and interstitial fibrosis, repressing renal inflammation. Western blot results showed that the NLRP3/ASC/Caspase-1 signaling pathway-related proteins decreased after TA treatment. In addition, TA also ameliorated impairments in the intestinal mucosa barrier and restored the expressions of intestinal tight junction proteins (Claudin-1, Occludin and ZO-1). Subsequently, it reduced LPS levels of DN mice in fecal and serum. Furthermore, 16S rRNA high-throughput sequencing showed that TA modulated gut microbiota dysbiosis and decreased the abundance of Gram-negative bacteria (Proteobacteria and *Escherichia-Shigella*).

**Conclusion:**

This study suggested that TA might exert a beneficial effect on renal interstitial fibrosis in DN mice by modulating gut microbiota dysbiosis, ameliorating impairments in the intestinal mucosa barrier, reducing the production and release of LPS, inhibiting the activation of NLRP3/ASC/Caspase-1 signaling pathway, and repressing renal inflammatory.

## 1 Introduction

Diabetic nephropathy (DN) is one of the most serious microvascular complications of diabetes mellitus, which is the leading cause of end-stage renal disease (ESRD) (Fox et al., 2012). The etiology and pathogenesis of DN are complicated and have not been fully elucidated. Interstitial fibrosis and glomerulosclerosis are typical pathological characteristics of DN. Renal interstitial fibrosis is defined by excessive extracellular matrix (ECM) accumulation and is associated with a decreased kidney function (Eddy, 2000). Increased inflammation is a major culprit of renal fibrosis development, but the mechanism of how inflammation starts is still un-known (Kumawat and Kaur, 2023). Recent studies have shown that gut microbiota dysbiosis is related to the occurrence and development of inflammation in DN (Feng et al., 2019; Zhao et al., 2019).

Gut microbiota dysbiosis enables leakage of proinflammatory bacterial products, which contribute to renal inflammation and interstitial fibrosis (Ramezani and Raj, 2014; Evenepoel et al., 2016). The increased abundance of Gram-negative bacteria in DN patients led to excessive production of lipopolysaccharide (LPS), which urther damages the mechanical barrier formed by intestinal epithelial cells and the tight junction between them (Linh et al., 2022). Studies have shown that LPS can enter the blood through the damaged intestinal mucosa and initiate renal inflammatory in DN (Fritsche, 2015). In addition, studies have shown that the NLRP3/ASC/Caspase-1 signaling pathway can be induced and activated by the pro-inflammatory mediator LPS, and the secretion of pro-inflammatory cytokines interleukin-1β (IL-1β) and interleukin-6 (IL-6) promotes cell death and induces automatic defense and inflammatory responses (Faustino Viviane et al., 2018; Andrade-Oliveira et al., 2019; Wang et al., 2022).

At present, the American Diabetes Association recommends the application of ACEIs, ARBs, SGLT2 inhibitors, GLP-1 agonists, mineralocorticoid receptor antagonists, and endothelin antagonists to slow the progression of renal disease and prevent or delay the onset of ESRD (Hu Q. et al., 2023). Clinical evidence has confirmed that long-term or excessive use of these drugs may lead to adverse reactions, such as hyperkalemia or hypokalemia, increasing cardiovascular risk and mortality (Pelle et al., 2022). Therefore, the bioactive components of non-toxic or low-toxic natural plants have attracted more and more attention. Thonningianin A (TA) is one of the main components of the natural medicine Penthorum chinense Pursh (PCP) (Wang et al., 2020). Modern pharmacological studies have confirmed that TA has anti-oxidation, anti-inflammatory and anti-fibrosis effects (Hu J. et al., 2023). However, there is no report on the anti-fibrotic effect of TA on DN mice, and the role of gut microbiota in the therapeutic efficacy of TA in DN remains to be further investigated.

In this study, a DN mouse model was established by STZ injection and treated with TA. Firstly, the therapeutic, anti-fibrotic and anti-inflammatory effects of TA on DN were evaluated. Secondly, the effects of TA on impairments in the intestinal mucosa barrier and LPS level were evaluated and Western blot was used to detect the changes of NLRP3/ASC/Caspase-1 signaling pathway-related proteins in kidney. Finally, 16S rRNA high-throughput sequencing was used to detect alteration of gut microbiota diversity and abundance in mice after TA treatment. This can help to elucidate the mechanism of TA in ameliorating renal interstitial fibrosis by modulating gut microbiota dysbiosis, ameliorating impairments in the intestinal mucosa barrier, reducing LPS release into blood, inhibiting NLRP3/ASC/Caspase-1 signaling pathway, and repressing renal inflammation in DN mice.

## 2 Materials and methods

### 2.1 Materials and reagents

Streptozotocin (STZ) (S0130-1G) was purchased from Sigma Chemical Co., Ltd. (St. Louis, MO, United States). TA was purchased from Shanghai Winherb Medical Science Co., Ltd. (Shanghai, China). ELISA kits for IL-6 (item number: E-MSEL-M0001) and IL-1β (item number: E-MSEL-M0003) were purchased from Wuhan Elaite Biotechnology Co., Ltd. (Wuhan, China). Lipopolysaccharide (LPS) (Cat No. 21100201) was purchased from Tianjin Zhengdao Biotechnology Co., Ltd. (Tianjin, China). Tissue lysates were prepared using a radioimmunoprecipitation assay (RIPA) lysis buffer (CWBIO) containing a protease/phosphatase inhibitor mixture. The following primary antibodies were used: E-cadherin (1:20,000, rabbit, 20874-1-AP, Proteintech, United States), α-SMA (1:1,000, rabbit, 14395-1-AP, Proteintech, United States), NLRP3 (1:500, rabbit, CAT # YT5382, ImmunoWay Biotechnology, United States), ASC (1:1,000, rabbit, 67824T, Cell Signaling Technology, United States), Caspase-1 (1:2,000, 22915-1-AP, Proteintech, United States), anti-ZO-1 (1:2,000, rabbit, 21773-1-AP, Proteintech, United States), Occludin (1:5,000, rabbit, 27260-1-AP, Proteintech, United States) and claudin-1 (1:2,000, rabbit, ab211737, Abcam, United Kingdom), β-actin (1:500, mouse, ab8226, Abcam, United Kingdom). The corresponding horseradish peroxidase (HRP) -conjugated secondary antibodies (7074V and 7076V) were purchased from CST (United States).

### 2.2 Animals and treatments

**FIGURE 1:**
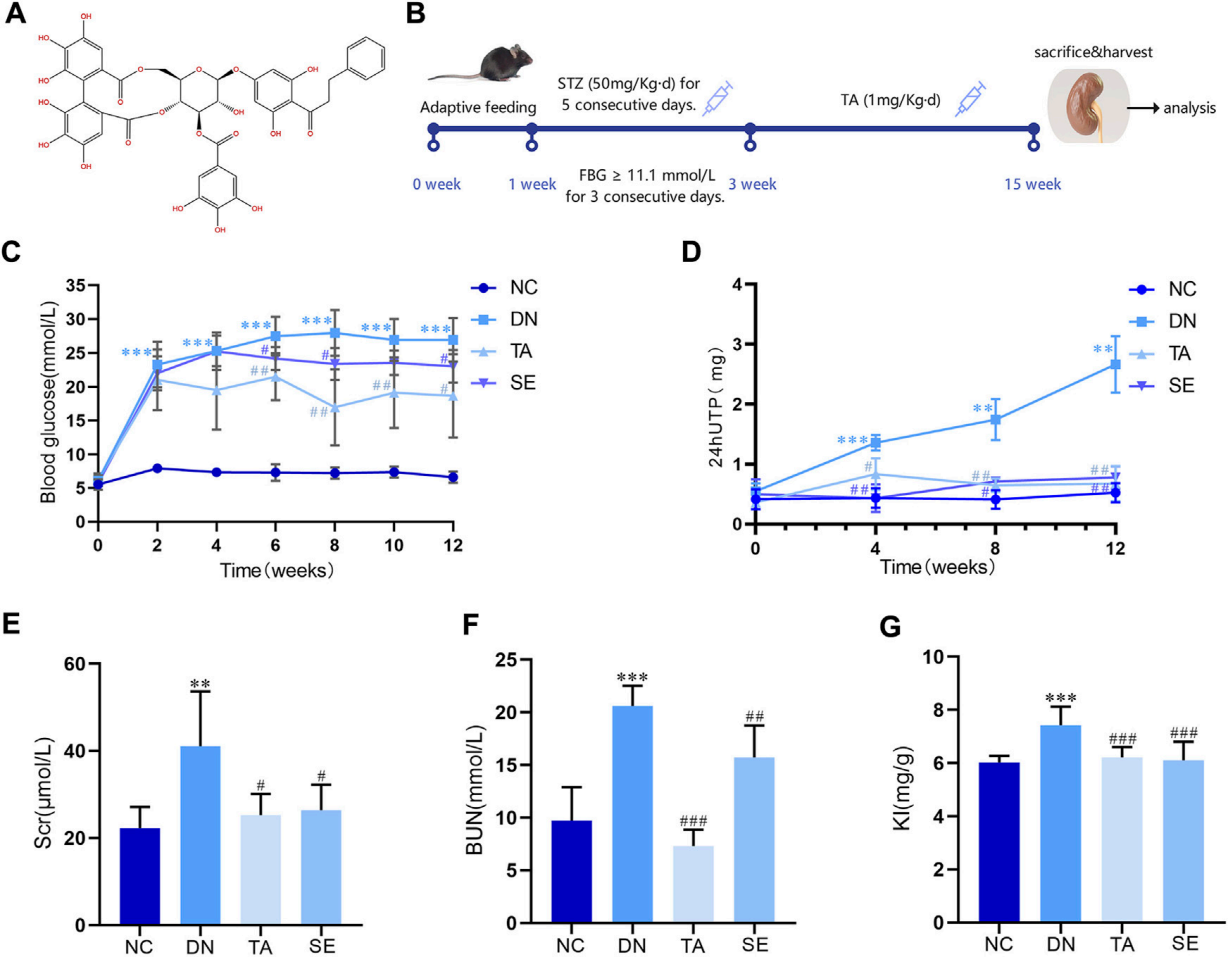
Thonningianin A (TA) improved body parameters in DN mice. **(A)** The molecular structure of thonningianin A (TA); **(B)** Flow diagram of the study; **(C)** Glu (n = 10); **(D)** 24hUTP (n = 5); **(E)** Scr; **(F)** BUN (n = 10); **(G)** KI (n = 10). Data are presented as mean ± SEM, **P < 0.01, ***P < 0.001, DN vs. NC; ^#^ *P* < 0.05, ^##^ *P* < 0.01, ^###^ *P* < 0.001, TA, SE vs. DN.

A total of 40 C57BL/6 male mice with average body weight 20 g were purchased from Beijing Vital River Laboratory Animal Technology Co., Ltd. (SCXK-2023-0011). The housing environment was maintained at 25°C ± 2°C, relative humidity was 50% ± 10%, light-dark cycle was 12 h/12 h, and animals were given ad libitum access to food and water. The experimental design is shown in Figure 1B. Mice were randomly divided into four groups (10 mice for each group): negative control (NC) group, DN group, TA group, and Semaglutide (SE) group. After 1 week of adaptive feeding, then DN, TA and SE groups were intraperitoneally injected with STZ (50 mg/kg, dissolved in 0.01 M sodium citrate buffer, pH: 4.2) for five consecutive days to induce diabetic nephropathy (Du et al., 2023). NC group was given equal citrate buffer as controls. The blood glucose (Glu) level of the mice was stable after a week. Subsequently, blood was taken from the mouse tail veins, and the level of fasting blood glucose (FBG) was measured. And FBG level >11.1 mmol/L for three consecutive days was considered to indicate DN (Yuan et al., 2019), and the mice were used for further research. TA group was given 0.1 mg/kg/d of TA, and SE group was given 40ug/kg/3d of SE for 12 weeks, while NC group and DN group were given equal normal saline during this time. The dosages of TA were referred to Sun et al. (2020). The dosages of SE were referred to Cardoso et al. (2023). The body weight and FBG of mice were measured every 2 weeks, and 24 h urine was collected every 4 weeks. Serum creatinine (Scr) and blood urea nitrogen (BUN) were tested at 12 weeks after treatment. At the end of the study, mice were fasted overnight, sacrificed by cervical dislocation. The blood samples were collected from the eyeballs and centrifuged (3,000 r/min, 15 min) to obtain serum for biochemical analysis. Subsequently, the kidney tissues were rapidly harvested and weighed to calculate kidney-to-body weight ratio (kidney index, KI). The renal and colonic tissues were used for Western blot, immunohistochemical assay, histologic examinations and other biochemical analysis. All serum and tissues samples were cryopreserved at −80°C. Colonic content was collected for the detection of LPS and microorganisms. All experimental procedures were approved by the Animal Ethics Committee of Beijing University of Chinese Medicine (Permission BUCM-2023042003-2110).

### 2.3 Biochemical parameter analysis

Levels of Scr and BUN were measured with commercial kits purchased from the Nanjing Jiancheng Bioengineering Institute (Nanjing, China). For the 24 h urinary protein (24hUTP) concentration, metabolic cages were used to record the 24 h total urine volume and collect 24 h urine samples from all mice. Then the supernatant of urine samples were collected to determine the 24hUTP levels using colorimetric methods as described previously.

### 2.4 Pathology staining

Kidney and colon tissues were fixed in 4% paraformaldehyde for 48 h, followed by a sequential process of dehydration through an ethanol gradient, clearance in xylene, immersion in wax, and embedding in paraffin, to prepare sections with a thickness of 3 μm. After deparaffinization and rehydration, the tissue sections were stained according to the instructions provided in the staining kit using Hematoxylin and Eosin (H&E), Periodic Acid-Schiff (PAS), or Masson’s Trichrome (MASSON) stains. Pathological alterations in the renal and colon tissues were examined under a light microscope, and representative images were captured for further analysis.

### 2.5 Immunohistochemistry

Following deparaffinization, antigen retrieval, and inhibition of endogenous peroxidase activity, the 3 μm-thick paraffin-embedded kidney tissue sections were incubated with primary antibodies against IL-1β (1:2,000, ab32362, Abcam, United Kingdom) and IL-6 (1:1,000, ab216341, Abcam, United Kingdom), and Colon tissue sections were incubated with primary antibodies against ZO-1 (1:2,000, rabbit, 21773-1-AP, Proteintech, United States), occludin (1:5,000, rabbit, 27260-1-AP, Proteintech, United States) and claudin-1 (1:2,000, rabbit, ab211737, Abcam, United Kingdom), at 4°C overnight.

### 2.6 Enzyme linked immunosorbent assay

After 12 weeks of TA intervention, renal levels of interleukin-1β (IL-1β) and interleukin-6 (IL-6) were measured in each group using ELISA kits (Elabscience, Wuhan, China), which was performed according to the manufacturers’ instructions.

### 2.7 Immunofluorescence staining

Kidney tissue sections were incubated with primary antibodies including NLRP3 (1:100, rabbit, # YT5382, ImmunoWay Biotechnology, United States), ASC (1:400, rabbit, 67824T, Cell Signaling Technology, United States), Caspase-1 (1:100, 22915-1-AP, Proteintech, United States) at 4°C overnight. The corresponding fluorescent secondary antibody was added and incubated at room temperature for 1 h. Dihydrochloride (DAPI) was added and incubated for 5 min. All sections were imaged using a laser scanning confocal microscope (Olympus, Tokyo, Japan). Semi-quantitative statistical analysis was performed using ImageJ 1.48 version (National Institutes of Health, Bethesda, MD, United States) based on six fields of view.

### 2.8 Western blot analysis

Kidney and colon tissue samples were mechanically disrupted and lysed using RIPA lysis buffer (Applygen, Beijing, China) to facilitate protein extraction. Following centrifugation, the supernatant containing the proteins was harvested. The protein concentration was subsequently determined through a BCA protein assay, followed by heat denaturation in the presence of a loading buffer. Equal volumes of the protein samples (10 μL per well) were loaded onto an SDS-PAGE gel for electrophoretic separation. After the separation process, the proteins were transferred onto a nitrocellulose membrane utilizing the wet transfer method. The membrane was then blocked with 5% skim milk and subsequently incubated overnight at 4°C with primary antibodies, including E-cadherin, α-SMA, NLRP3, ASC, Caspase-1, ZO-1, occludin, claudin-1 and β-actin. Post incubation, the membrane was washed with TBST to remove unbound antibodies, before incubation with HRP-conjugated secondary antibodies (either anti-rabbit or anti-mouse IgG) for 1 h. The relative expression of target proteins was calculated using β-actin as an internal reference. ImageJ software was employed to analyze the densitometric values of the bands and to calculate the relative expression of the proteins of interest.

### 2.9 Determination of LPS levels in serum and fecal samples

The collected whole blood samples were placed at room temperature for 2 h and centrifuged at 1,000 xg for 20 min. The supernatant was taken and the serum LPS content was determined using a LPS ELISA kit. The collected fecal samples (greater than 50 mg) were washed three times with phosphate buffered saline (PBS), and the precipitate was collected by centrifugation and heavy. PBS buffer (9 mL PBS buffer per Gram of feces) was added, and they were crushed with 4,000 g ultrasound for 10 min. The supernatant was taken and the fecal LPS content was measured using the LPS ELISA kit.

### 2.10 16S rRNA high-throughput sequencing analysis of gut microbiota

DNA was extracted from mouse cecum feces using the HiPure Soil DNA Kit (Magen, Guangzhou, China), and the purity and concentration of the extracted DNA were determined with 2% agarose gel electrophoresis. The primer sequences 341F (5′-CCTACGGGNGGCWGCAG-3′) and 806R (5′-GGACTACHVGGGTATCTAAT-3′) were used to amplify the V3-V4 hyper-variable region of the bacterial 16S rRNA gene (45). The samples were then sequenced in parallel utilizing Illumina DNA Prep Kit (Illumina, CA, United States) according to the user manual. The resulting raw data files were manipulated and filtered with the QIIME (version 1.9.1) software package. Raw sequences were imported into FASTP (version 0.18.0), and FLASH software (version 1.2.11) was used for pair-end double-end sequence splicing and screening for sequence optimization. Sequences with >97% similarity were clustered and annotated to generate operational taxonomic units (OTUs) using UPARSE software (version 9.2.64). Alpha diversity and microbial taxon distribution analyses were performed with QIIME software.

### 2.11 Statistical analysis

All data were reported as mean ± standard error of the mean (SEM). One-way analysis of variance (ANOVA) followed by post hoc Fisher’s least significant difference (LSD) and post hoc Tamhane’s test was used in assessing the differences between the two groups. All statistical analyses were performed using SPSS 20 software (SPSS Inc., Chicago, IL, United States). *P* < 0.05 was considered statistically significant.

## 3 Results and analysis

### 3.1 TA improves physical and biochemical indicators of in DN mice

Figure 1A is the molecular structure of thonningianin A. Figure 1B shows the flow diagram of the study. In this research, we established a DN mice model by continuous intraperitoneal injection of low-dose STZ. As shown in Figure 1C, Glu of the DN group exceeded 11.1 mmol/L for three consecutive days, a significant increase compared to the NC group, while SE treatment significantly reduced Glu, confirming the success of the modeling. And TA dramatically mitigates Glu (*p* < 0.05). DN is characterized by persistent microalbuminuria, a decreased glomerular filtration rate (GFR) and increased urine albumin excretion. Urinary protein and microalbuminuria are sensitive indicators of glomerular dysfunction and tubular impairment. As shown in Figure 1, as compared to the NC group, the DN mice exhibited remarkable increases in the levels of 24hUTP (Figure 1D), Scr(Figure 1E), BUN(Figure 1F) and KI(Figure 1G). In particular, TA supplementation greatly downregulated the levels of 24hUTP (Figure 1D), Scr(Figure 1E), BUN(Figure 1F), and KI (Figure 1G) (*p* < 0.05). According to the results, TA markedly mitigated blood glucose, decreased 24-h urinary total protein, improved renal dysfunction and KI in DN mice.

### 3.2 TA ameliorated renal injury and interstitial fibrosis in DN mice

**FIGURE 2:**
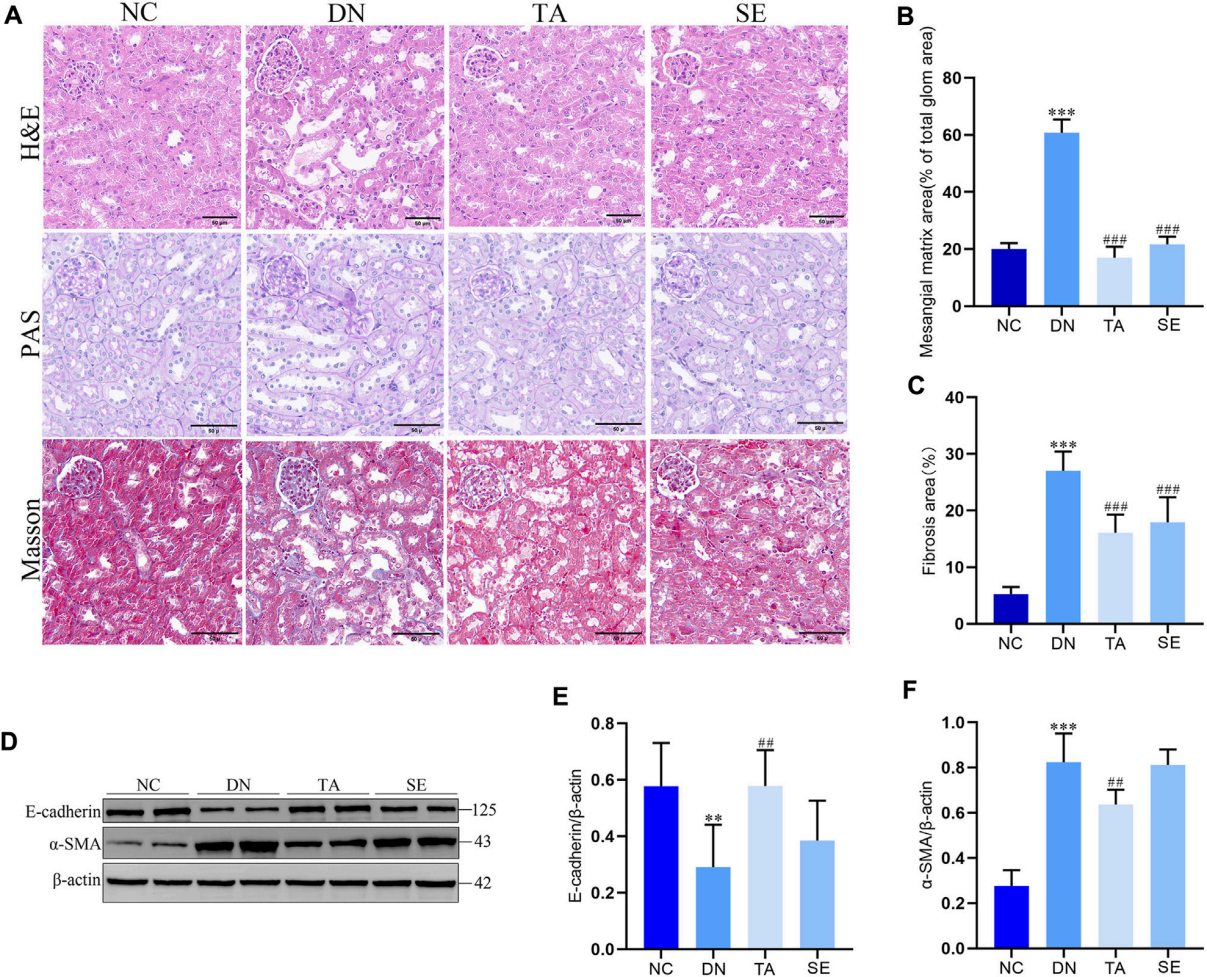
TA attenuated renal injury and interstitial fibrosis. **(A)** Pathological changes in representative kidney sections from each group of mice by H&E staining (original magnifications, ×400), PAS-staining (original magnifications, ×400), and Masson’s trichrome staining (original magnifications, ×400); **(B)** Mesangial matrix area; **(C)** Fibrosis area; **(D)** Representative Western blots showing the detection of E-cadherin and α-SMA; **(E)** Relative protein expression of E-cadherin; **(F)** Relative protein expression of α-SMA. Data are presented as mean ± SEM, n = 5. **P < 0.01, ***P < 0.001, DN vs. NC; ^#^ *P* < 0.05, ^##^ *P* < 0.01, ^###^ *P* < 0.001, TA, SE vs. DN.

We further explored the effect of TA on renal injury and interstitial fibrosis using the representative images from renal sections as stained for hematoxylin–eosin (H&E), PAS and Masson. As shown in Figure 2A, HE staining revealed significant pathological changes in the DN group compared to the NC group, including glomerular hypertrophy, enlarged Bowman’s capsule space, and renal tubular dilation, along with vacuolar degeneration, swelling, or desquamation of renal tubular epithelial cells. Masson’s trichrome staining revealed intact glomeruli, renal tubules, and interstitium in the NC group, with only weak blue staining observed. In contrast, the DN group demonstrated a substantial increase in blue-stained areas within the kidney tissue, indicating an increased degree of fibrosis. PAS staining revealed that compared with the NC group, the mesangial region in the DN group was significantly widened, the mesangial matrix was proliferated (Figure 2B) and the Glomerular basement membrane was thickened. Overall, TA significantly improved various renal pathological lesions of the above-mentioned DN mice (Figure 2C). In addition, E-cadherin expression in the DN group was significantly decreased, while α-SMA expression was significantly increased compared with the NC group (Figure 2D). TA significantly increased E-cadherin (Figure 2E) expression and reduced α-SMA (Figure 2F) expression in DN mice (*p* < 0.01). The above results indicate that TA treatment effectively protected against renal injury, especially renal interstitial fibrosis. Overall, the results described above indicated that TA could alleviate renal injury and interstitial fibrosis in the DN mice.

### 3.3 TA repressed renal inflammation in DN mice

**FIGURE 3:**
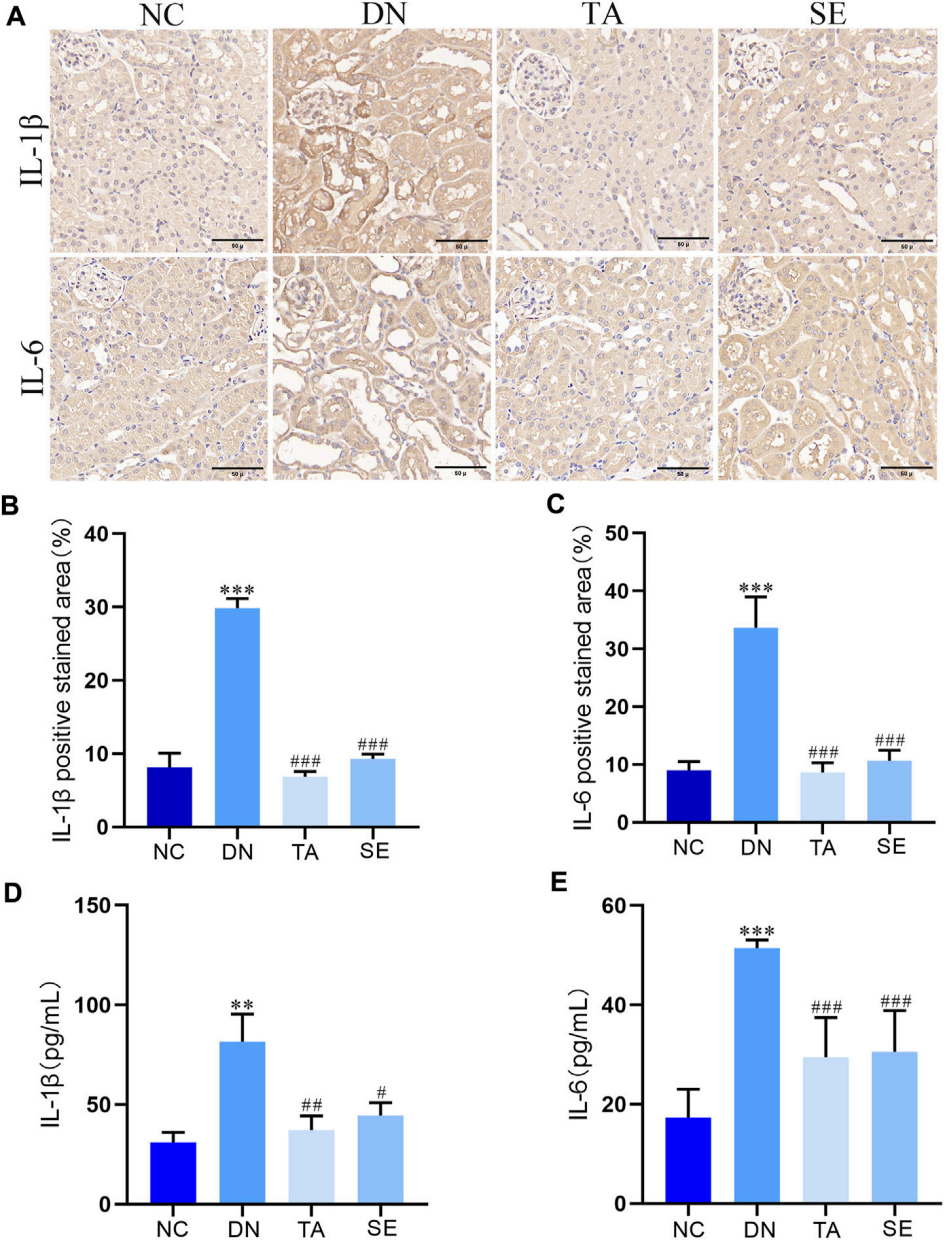
TA repressed the renal inflammation in DN mice. **(A)** Expression level of IL-1βand IL-6 in kidney tissue was detected by immunohistochemistry (original magnifications, ×400); **(B)** Relative IL-1β expression examined by immunohistochemistry was analyzed and shown in histogram; **(C)** Relative IL-6 expression examined by immunohistochemistry was analyzed and shown in histogram.; **(D)** ELISA analysis indicated that the expression levels of IL-1β in kidney tissue; **(E)** ELISA analysis indicated that the expression levels of IL-6 in kidney tissue. Data are presented as mean ± SEM, n = 5. **P < 0.01, ***P < 0.001, DN vs. NC; ^#^ *P* < 0.05, ^##^ *P* < 0.01, ^###^ *P* < 0.001, TA, SE vs. DN.

We further explored whether TA alleviated kidney injury and fibrosis is associated with mitigating renal inflammation by detecting the levels of IL-6 and IL-1β in kidney tissue of mice in each group. As shown in Figure 3A, immunohistochemistry analysis exhibited that little staining was observed in normal kidney tissue. Conversely, as compared with the NC group, the DN group exhibited strong and diffuse immune expression of IL-1β (Figure 3B) and IL-6 (Figure 3C) in the kidney tissue sections (*p* < 0.001), while the intensity of staining was significantly decreased with low immune expression when treated with TA (*p* < 0.001). ELISA analysis showed that the levels of IL-1β (Figure 3D) and IL-6 (Figure 3E) in DN group were significantly increased (*p* < 0.01), while the levels of IL-1β and IL-6 in TA group were significantly decreased (*p* < 0.01). The above results showed that TA repressed renal inflammation by regulating the inflammatory mediators in DN mice.

### 3.4 TA downregulated the NLRP3/ASC/Caspase-1 signaling pathway

**FIGURE 4:**
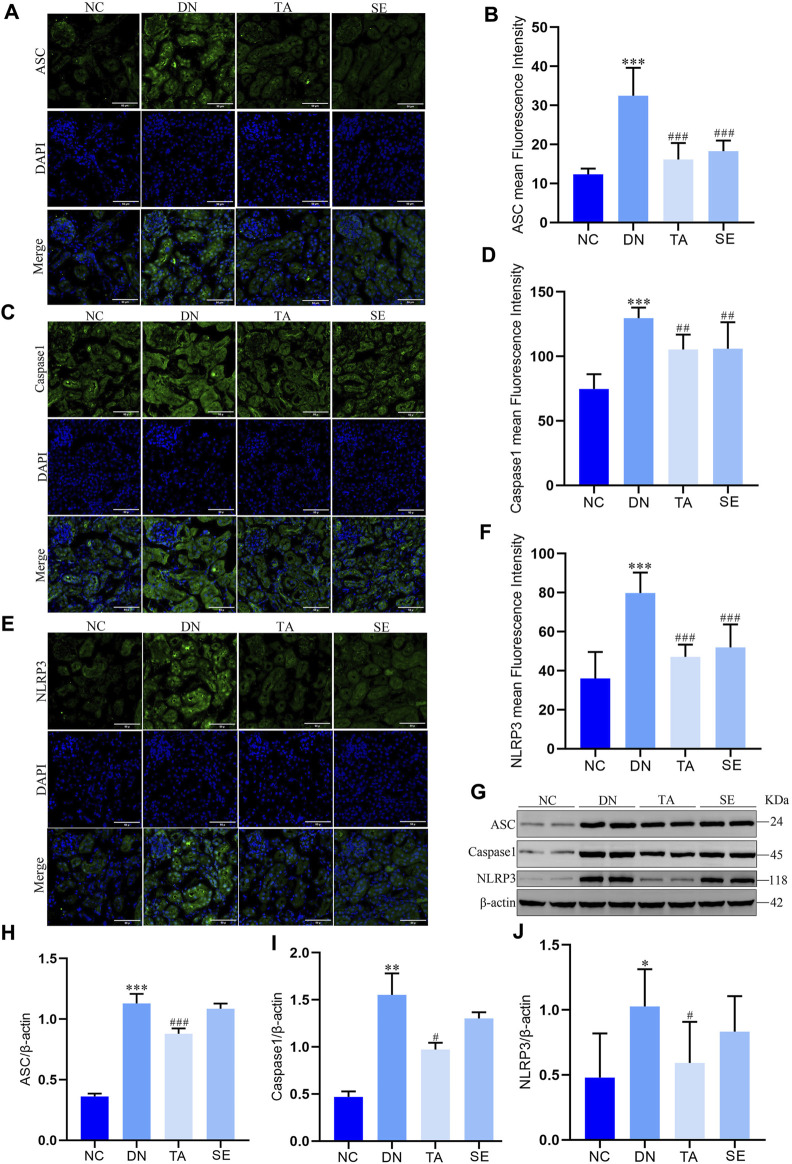
TA downregulated NLRP3/ASC/Caspase-1 signaling pathway. **(A)** Immunofluorescence observation and semiquantitative analysis of the expression level of mouse kidney ASC protein (original magnifications, ×400); **(B)** ASC mean Fluorescence Intensity in mouse kidney; **(C)** Immunofluorescence observation and semiquantitative analysis of the expression level of mouse kidney Caspase-1 protein (original magnifications, ×400); **(D)** Caspase-1 mean Fluorescence Intensity in kidney; **(E)** Immunofluorescence observation and semiquantitative analysis of the expression level of mouse kidney NLRP3 protein; **(F)** NLRP3 mean Fluorescence Intensity in mouse kidney; **(G)** Western blot assay of NLRP3, ASC, and Caspase-1 expression in kidney tissue; **(H)** Relative protein expression of ASC; **(I)** Relative protein expression of Caspase-1; **(J)** Relative protein expression of NLRP3. Data are presented as mean ± SEM, n = 5. **P < 0.01, ***P < 0.001, DN vs. NC; ^#^ *P* < 0.05, ^##^ *P* < 0.01, ^###^ *P* < 0.001, TA, SE vs. DN.

It has been confirmed that the NLRP3/ASC/Caspase-1 signaling pathway is activated in DN, thus contributing to the occurrence of renal inflammation (Ding et al., 2018; Al Mamun et al., 2021; Yang et al., 2021). Immunofluorescence staining and Western blotting were conducted to investigate the molecular mechanisms underlying the alleviation effect of TA on renal interstitial fibrosis and kidney inflammatory responses. Immunofluorescence staining and semiquantitative analysis showed that the expression of ASC (Figures 4A, B), Caspase-1 (Figures 4C, D) and NLRP3 (Figures 4E, F) in the kidneys of mice in the DN group was significantly increased compared with that in the NC group, indicating that inflammation and pyroptosis were accompanied by the process of kidney injury in DN. However, the process was reversed after treatment with TA. Moreover, protein levels were also evaluated by Western blotting of kidney tissue, and a similar trend was seen, as shown in Figures 4G–J. Taken together, these results provided evidence that TA could repressed renal inflammatory by suppressing the NLRP3/ASC/Caspase-1 signaling pathway that were activated in the kidneys of DN mice.

### 3.5 TA ameliorated impairments in the intestinal mucosa barrier and reduced fecal and serum LPS levels in DN mice

**FIGURE 5:**
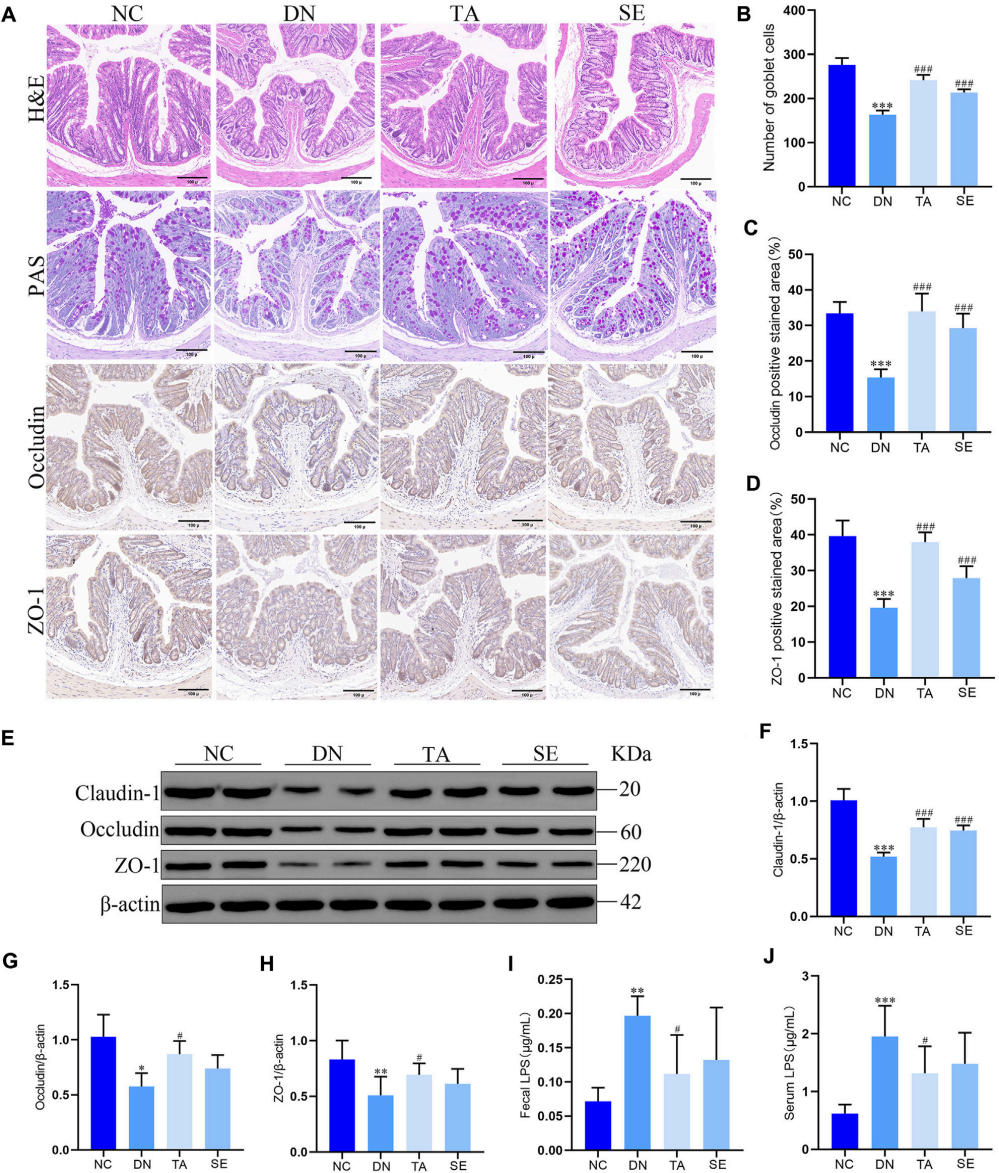
Effects of TA on the intestinal mucosa barrier and LPS levels in DN mice. **(A)** Pathological assessment of H&E-stained and PAS-stained mice colonic sections; Expression of Occludin and ZO-1 in colon tissues was detected by immunohistochemistry (original magnifications, ×200); **(B)** Number of goblet cells; **(C)** Statistical analysis of Occludin by immunohistochemical staining; **(D)** Statistical analysis of ZO-1 by immunohistochemical staining; **(E)** Western blot assay of Claudin-1, Occludin, and ZO-1 expression in colon tissue; **(F)** Relative protein expression of Claudin-1; **(G)** Relative protein expression of Occludin; **(H)** Relative protein expression of ZO-1; **(I)** Fecal LPS levels in DN mice; **(J)** Serum LPS levels in DN mice. Data are presented as mean ± SEM, n = 5. **p < 0.01, ***p < 0.001, DN vs. NC; ^#^ *P* < 0.05, ^##^ *P* < 0.01, ^###^ *P* < 0.001, TA, SE vs. DN.

In order to further explore whether the inhibition of NLRP3/ASC/caspase-1 signaling pathway by TA is related to intestinal mucosal barrier and LPS, we detected the colonic mucosal structure by HE and PAS staining, the expression of ZO-1, occludin and claudin-1 in the colon by immunohistochemistry and WB, and the levels of LPS in feces and serum. As shown in Figures 5A, B, the DN group showed thickening of the basal layer of the colonic crypt, branching of the colonic crypt, and atrophy of the colonic crypt, while the TA group showed significant improvement (*p* < 0.01). The TA administration also restored the expressions of intestinal tight junction proteins (Figures 5C, D), including Claudin-1 (Figure 5F), Occludin (Figure 5G), and ZO-1 (Figure 5H), which were decreased in the DN group. In addition, the level of LPS in feces (Figure 5I) and serum (Figure 5J) of DN group was significantly higher than that of the NC group, while TA showed a significant decrease (*p* < 0.05). The above results indicate that TA ameliorated impairments in the intestinal mucosa barrier and reduced the release of LPS into the blood in DN mice.

### 3.6 TA modulated gut microbiota dysbiosis in DN mice

**FIGURE 6:**
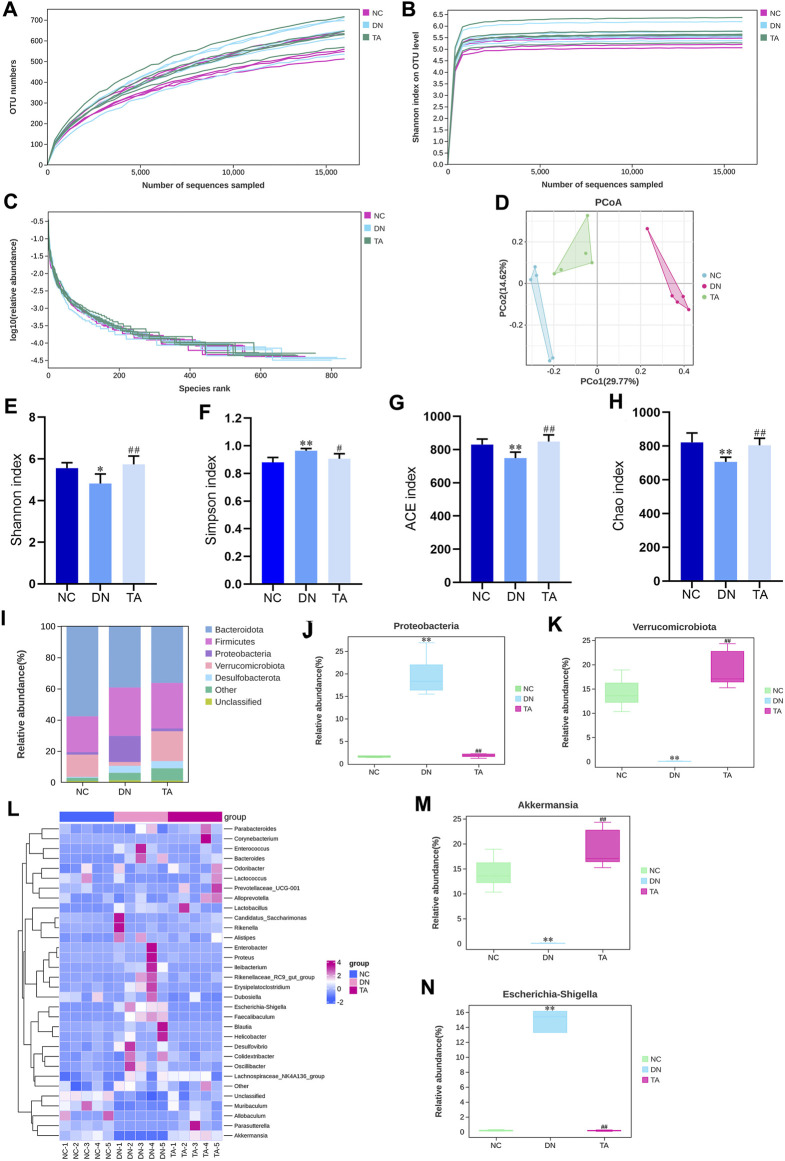
TA modulates DN-induced gut microbiota dysbiosis. **(A)** OTU numbers; **(B)** Shannon index on OTU; **(C)** log10 (relative abundance); **(D)** Unweighted UniFrac PCoA (principal coordinates analysis); **(E)** Shannon index; **(F)** Simpson index; **(G)** ACE index; **(H)** Chao index; **(I)** Relative abundance of intestinal microbiota at the phylum level, **(J)** Proteobacteria and **(K)** Verrucomicrobiota. **(L)** Abundance changes in representative bacteria at the genus level are shown in a heatmap. Genera that were statistically different with TA treatment: the relative abundance of **(M)** *Akkermansia* and **(N)** *Escherichia-Shigella* group. All data were expressed as the mean ± SD (n = 6). ^#^ *P* < 0.05, ^##^ *P* < 0.01, ^###^ *P* < 0.001 vs. NC group; *P < 0.05, **P < 0.01 vs. DN group. Data are presented as mean ± SEM, n = 5. **P < 0.01, ***P < 0.001, DN vs. NC; ^#^ *P* < 0.05, ^##^ *P* < 0.01, ^###^ *P* < 0.001, TA vs. DN.

To further investigate whether the antifibrotic effect of TA is related to the intestinal microbiot, we analyzed the microbiota in the feces of the mice in each group. 16S rRNA could determine the diversity and species richness of intestinal microorganisms in mice from different treatment groups (Figures 6A–D). The alpha diversity of the gut microbial community was assessed by calculating the Shannon (Figure 6E), Simpson (Figure 6F), ACE (Figure 6G) and Chao (Figure 6H) indices. Alpha diversity analysis revealed that the diversity and richness of faecal microbiota were considerably reduced in DN mice After TA treatment, the Chao, ACE and Shannon indices were significantly elevated, while the Simpson indices was significantly reduced, indicating that faecal microbiota diversity and richness increased (*P* < 0.05). Next, we analyzed the differences in beta diversity by principal coordinate analysis (PCoA). The PCoA results showed that the samples in the DN group were significantly separated from those in the NC group, while the samples in the TA group were distributed in a similar area to those in the NC group (Figures 6D). Furthermore, we analyzed the relative abundances of gut microbiota at the phylum and genus levels in each group. At the phylum level, *Firmicutes* and *Bacteroidetes* were the dominant taxa in gut microbiota at the phylum level for each group (Figures 6I). The Firmicutes/Bacteroidetes (F to B) ratio and the relative abundances of Proteobacteria (Figure 6J) was significantly higher in the DN group compared to the NC group, while it was decreased after TA treatment (*P* < 0.01). At the genus level, the relative abundances of Verrucomicrobiota (Figure 6K) and Akkermansia (Figures 6L, M) were significantly lower in the DN model compared, and the relative abundances of *Escherichia-shigella* (Figures 6L, N) was significantly higher, and to the NC group (*p* < 0.01). Compared to the DN group, the relative abundances of Verrucomicrobiota (Figure 6K) and *Akkermansia* (Figure 6M) were significantly increased, and the relative abundances of *Escherichia-shigella* (Figure 6N) significantly decreased in the TA group.

## 4 Discussion

**FIGURE 7:**
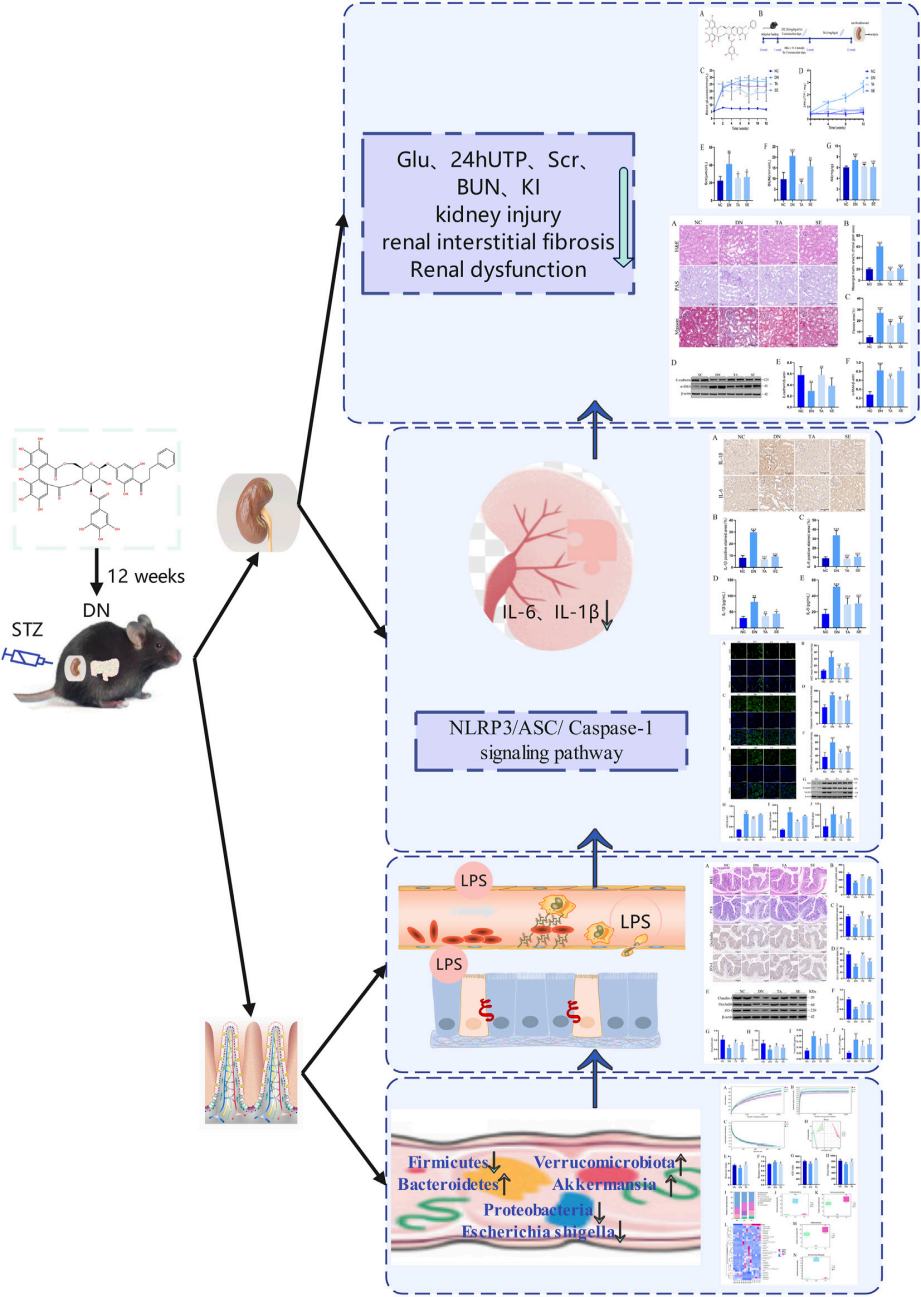
Summarizing figure of the mechanism of TA in ameliorating renal interstitial fibrosis in DN mice. Multiple effects of TA on gut microbiota, fecal and serum LPS levels, intestinal mucosa barrier, NLRP3/ASC/Caspase-1 signaling pathway, renal inflammation, renal injury and interstitial fibrosis and physical and biochemical indicators.

Diabetic nephropathy is the most serious microvascular complication of diabetes, with an important pathological feature of renal interstitial fibrosis (Lin et al., 2018). However, there is still a lack of effective measures against renal interstitial fibrosis to delay renal failure. Penthorum chinense Pursh (“Gan-Huang-Cao” or “Che-Gen-Cai” in Chinese; Penthoraceae) is widely used in China’s local and traditional medical systems and is also available as a vegetable or functional drinks (Chen et al., 2022). Thonningianin A (TA), a substance derived from PCP, significantly reversed the liver injuries, via antioxidation, oxidative stress, apoptosis, mitochondrial signaling pathways, and related geness (Wang et al., 2020). In the present study, we found that TA modulated gut microbiota dysbiosis and suppressed the inflammatory response may be a potential therapeutic strategy for the treatment of renal interstitial fibrosis. Through 16S rRNA analysis, we found that TA modulated gut microbiota dysbiosis and reduced the abundance of Gram-negative bacteria to reduce the production and release of LPS, thereby ameliorated impairments in the intestinal mucosa barrier, inhibited the NLRP3/ASC/Caspase-1 signaling pathway and further repressed the impact of inflammation on renal interstitial fibrosis in DN mice (Figure 7).

C57BL/6 mice are considered a good animal model for DN (Kitada et al., 2016). The DN group exhibited an enhancement of FBG and 24hUTP, an increased Scr, BUN, and KI, which could be significantly reduced by TA supplementation. Epithelial-mesenchymal transition (EMT) is the most important cause of renal interstitial fibrosis and is characterized by renal tubular epithelial cells that acquire mesenchymal phenotypes and myofibroblast functionss (Li et al., 2019). The transition of EMT induces kidney epithelial cells to decrease the expression of adherens junction proteins such as E-cadherin, and strongly induces the expression of fibroblast markers, including vimentin and α-smooth muscle actin (α-SMA) (Liu, 2011). In our study, renal interstitial fibrosis was aggravated in the DN group compared to the NC group, while TA treatment significantly ameliorated renal interstitial fibrosis. The expression of E-cadherin was significantly restored with TA, while α-SMA was remarkably downregulated compared with the DN group. The results further indicated that TA had beneficial anti-fibrotic effects, providing evidence with respect to the potential therapeutic impact on renal interstitial fibrosis.

The correlation between fibrosis and inflammation has been established and supported by morphological evidence. A large number of studies have shown the vital role of chronic inflammation in the initiation of the fibrotic response and the progression of fibrosiss in DN (Zheng and Zheng, 2016; Kriz et al., 2023). In our present study, the secretion and expression of IL-1β and IL-6 in kidney tissue of the TA group were remarkably downregulated. Studies have shown that the activation of NLRP3/ASC/Caspase-1 signaling pathway can lead to inflammatory response, the production of inflammatory cytokines and the recruitment of acute inflammatory cells mediated by chemokines, which is the upstream signaling pathway to regulate inflammation (Elliott and Sutterwala, 2015). Our results showed that the expression levels of NLRP3/ASC/Caspase-1 signaling pathway-related proteins in kidney tissue of DN mice were significantly increased, while significantly decreased after TA treatment. Therefore, it is believed that the activation of the NLRP3/ASC/Caspase-1 signaling pathway is a primary factor promoting renal micro-inflammation and interstitial fibrosis in DN mice. In our present study, TA had a beneficial effect on renal inflammation in DN mice by reducing the release of pro-inflammatory cytokines, along with suppressing the NLRP3/ASC/Caspase-1 signaling pathway, and further reduces renal interstitial fibrosis.

LPS is a component of Gram-negative bacteria to induce production of pro-inflammatory mediators. DN is accompanied by gut microbiota dysbiosis and an increase in the relative abundance of Gram-negative bacteria, which leads to a high level of production of LPS, and in turn damages the intestinal mucosal barrier and enters the blood circulation to reach the kidneys (Deng et al., 2022). A recent study has shown that LPS can activate the NLRP3/ASC/Caspase-1 signaling pathway, leading to renal inflammation and interstitial fibrosis (Ding et al., 2018; Wan et al., 2022). Our results showed that relative to the NC group, mice in the DN group had a significant increase in the relative abundance of Gram-negative bacteria (Proteobacteria and *Escherichia-shigella*), accompanied by impaired colonic mucosal barrier, decreased expression of intestinal tight junction proteins, and significantly increased LPS levels in feces and serum. In contrast, TA reduced the relative abundance of Gram-negative bacteria, repaired the intestinal mucosal barrier, elevated the expression of intestinal tight junction proteins, and decreased LPS levels in feces and serum. In addition, it has been demonstrated that decreased Bacteroidetes abundance and increased Firmicutes abundance were shown to be associated with low levels of inflammations (Turnbaugh et al., 2006). Another study showed that the abundance levels of Verrucomicrobiota and *Akkermansia* were positively correlated with intestinal barrier integrity and intestinal tight junction protein expression, but negatively correlated with serum LPS concentration (Barlow et al., 2015; Gryaznova et al., 2022). TA could increase the abundance of Bacteroidetes, Verrucomicrobiota, and *Akkermansia*, while decrease the abundance of Firmicutes.

In conclusion, our study is the first attempt to evaluate the protective effects of TA on DN and associated renal fibrosis by maintaining the gut microbiota homeostasis. The results indicated that TA could ameliorate renal interstitial fibrosis in DN mice through modulating gut microbiota dysbiosis, ameliorating impairments in the intestinal mucosa barrier, reducing the production and release of LPS, inhibiting the activation of NLRP3/ASC/Caspase-1 signaling pathway, and repressing renal inflammatory. Hence, as an adjuvant therapy, TA may be clinically valuable.

## Data Availability

The data presented in the study are deposited in the DRYAD repository, available at https://doi.org/10.5061/dryad.tht76hf7h.
