# Supplementary figures and images for: Thonningianin A ameliorated renal interstitial fibrosis in diabetic nephropathy mice by modulating gut microbiota dysbiosis and repressing inflammation
Source: Front Pharmacol. 2024 Aug 13;15:1389654. doi: 10.3389/fphar.2024.1389654 (PMC11347433; doi:10.3389/fphar.2024.1389654)

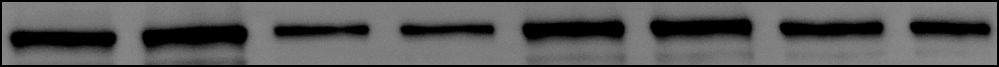

Supplement: Supplementary file 1 [file DataSheet1.ZIP › WB/Figure2/E-cadherin/E-cadherin-1-1.tif]

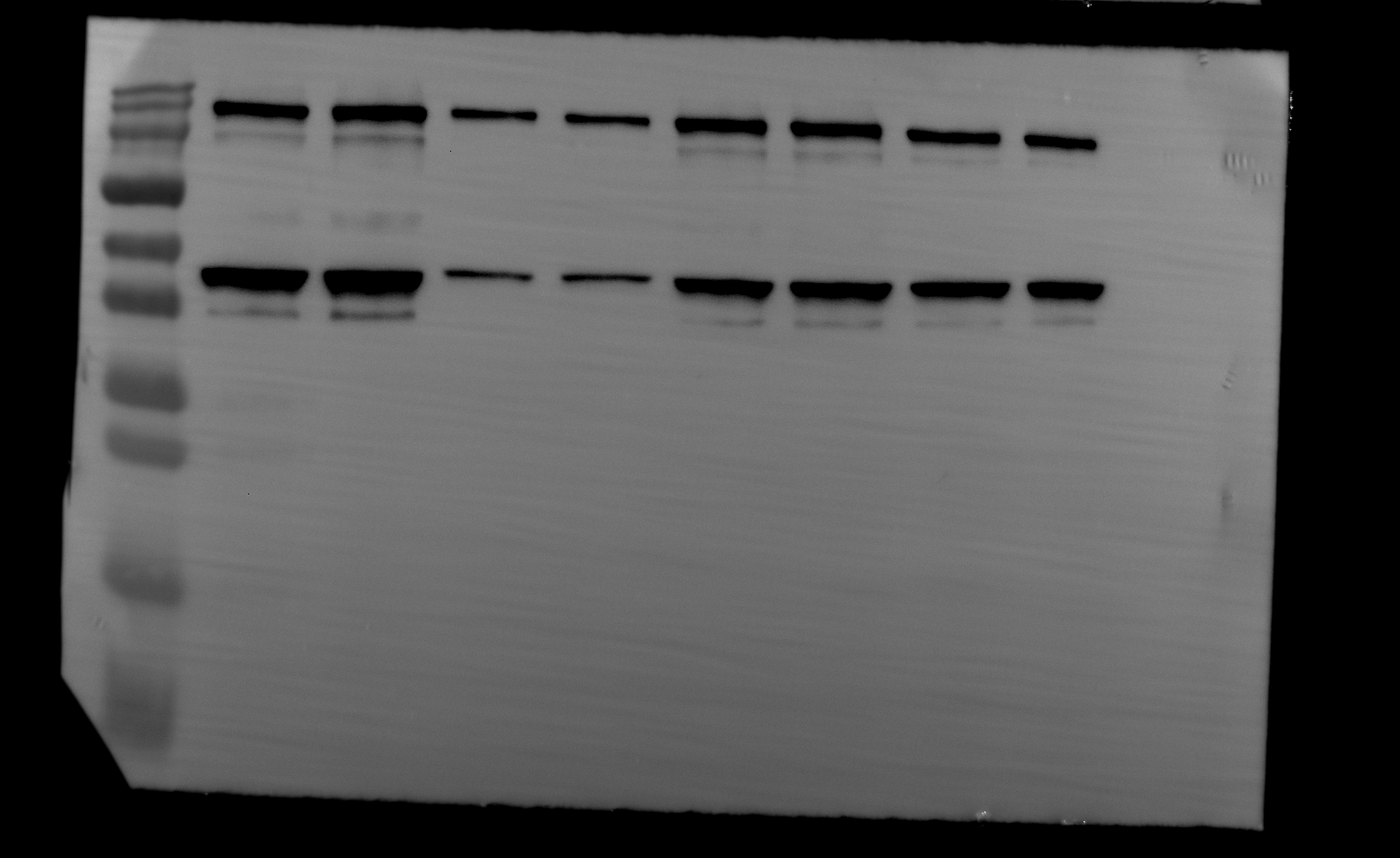

Supplement: Supplementary file 1 [file DataSheet1.ZIP › WB/Figure2/E-cadherin/E-cadherin-1.tif]

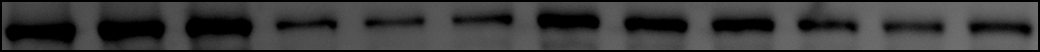

Supplement: Supplementary file 1 [file DataSheet1.ZIP › WB/Figure2/E-cadherin/E-cadherin-2-1.tif]

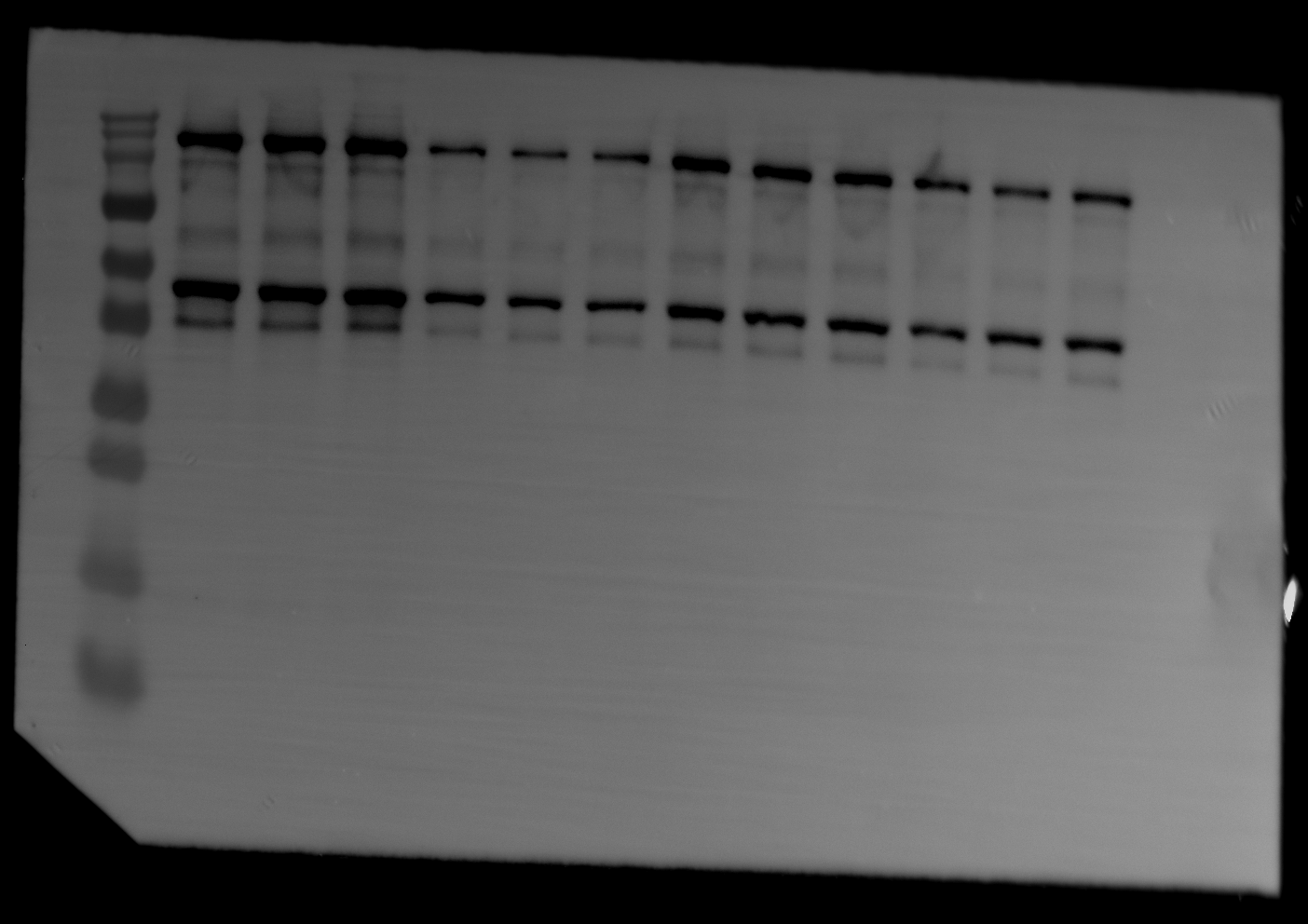

Supplement: Supplementary file 1 [file DataSheet1.ZIP › WB/Figure2/E-cadherin/E-cadherin-2.tif]

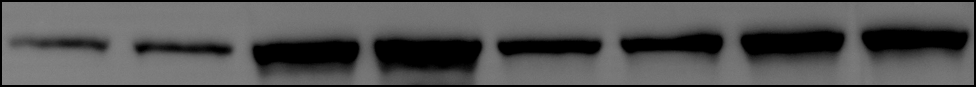

Supplement: Supplementary file 1 [file DataSheet1.ZIP › WB/Figure2/α-SMA/α-SMA 1-1.tif]

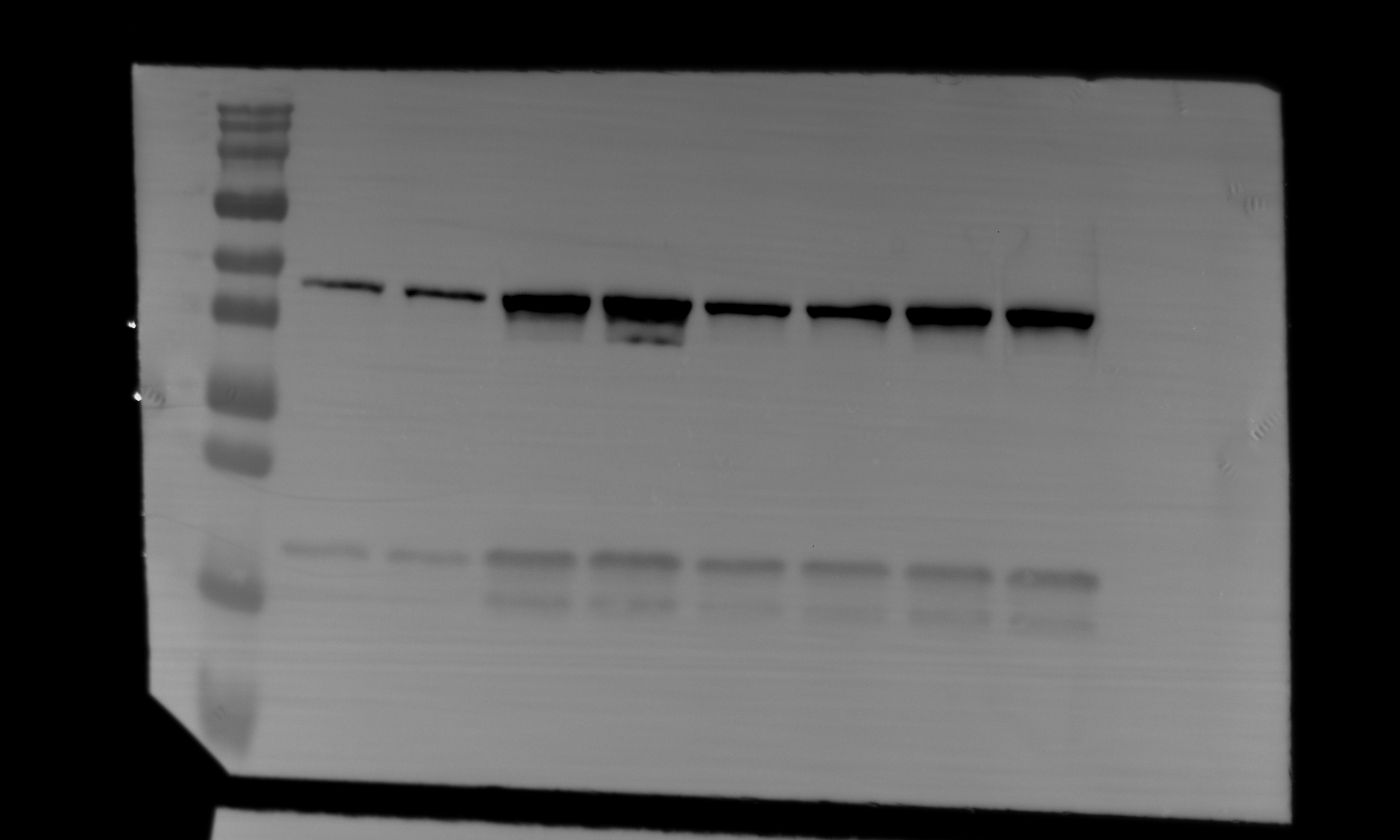

Supplement: Supplementary file 1 [file DataSheet1.ZIP › WB/Figure2/α-SMA/α-SMA 1.tif]

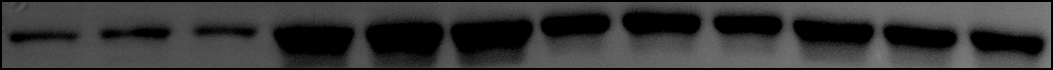

Supplement: Supplementary file 1 [file DataSheet1.ZIP › WB/Figure2/α-SMA/α-SMA 2-1.tif]

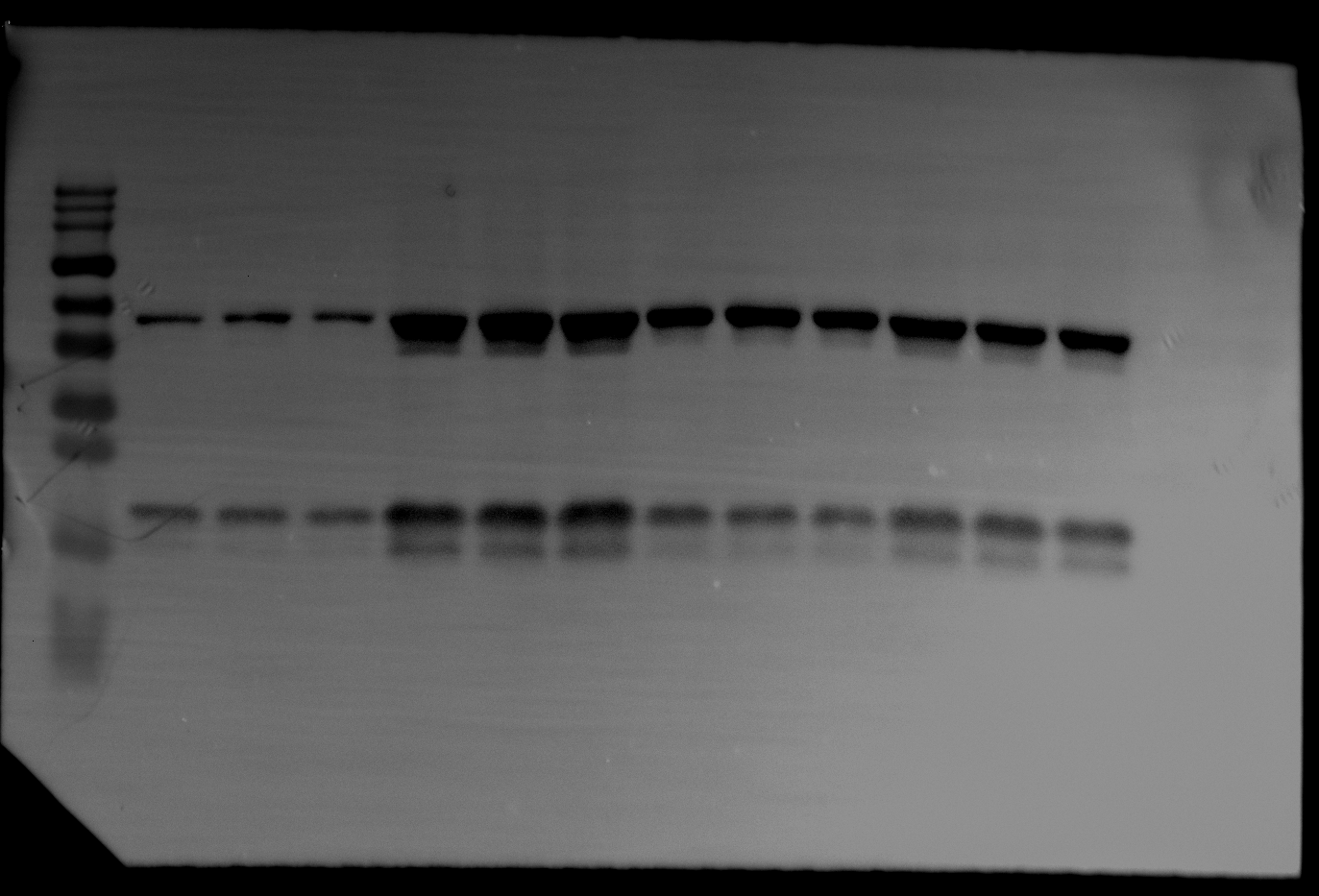

Supplement: Supplementary file 1 [file DataSheet1.ZIP › WB/Figure2/α-SMA/α-SMA 2.tif]

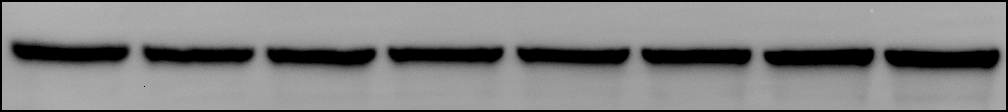

Supplement: Supplementary file 1 [file DataSheet1.ZIP › WB/Figure2/β-actin/β-actin 1-1.tif]

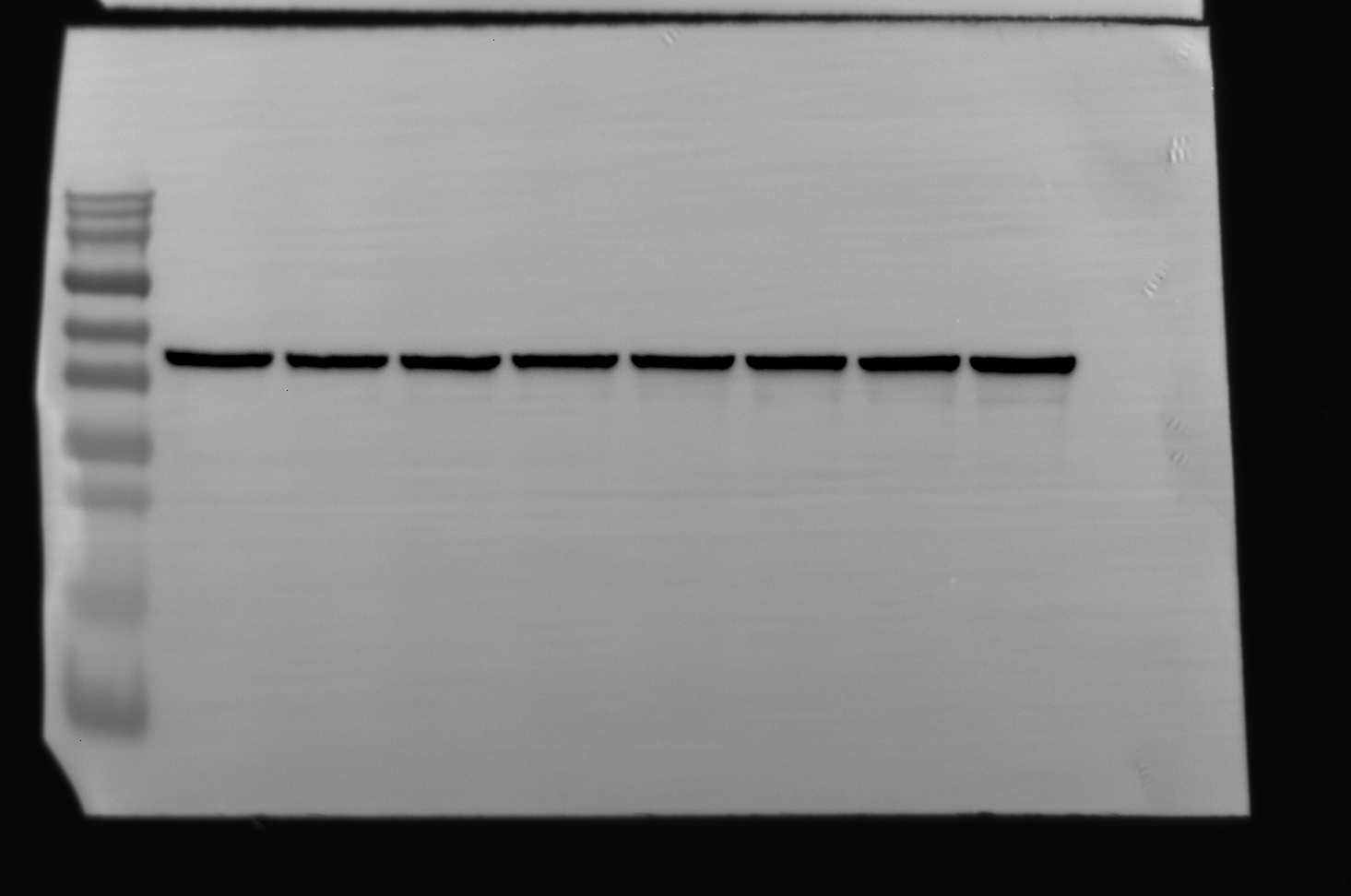

Supplement: Supplementary file 1 [file DataSheet1.ZIP › WB/Figure2/β-actin/β-actin 1.tif]

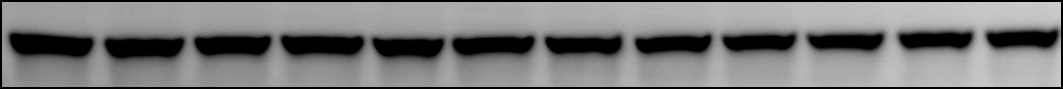

Supplement: Supplementary file 1 [file DataSheet1.ZIP › WB/Figure2/β-actin/β-actin 2-1.tif]

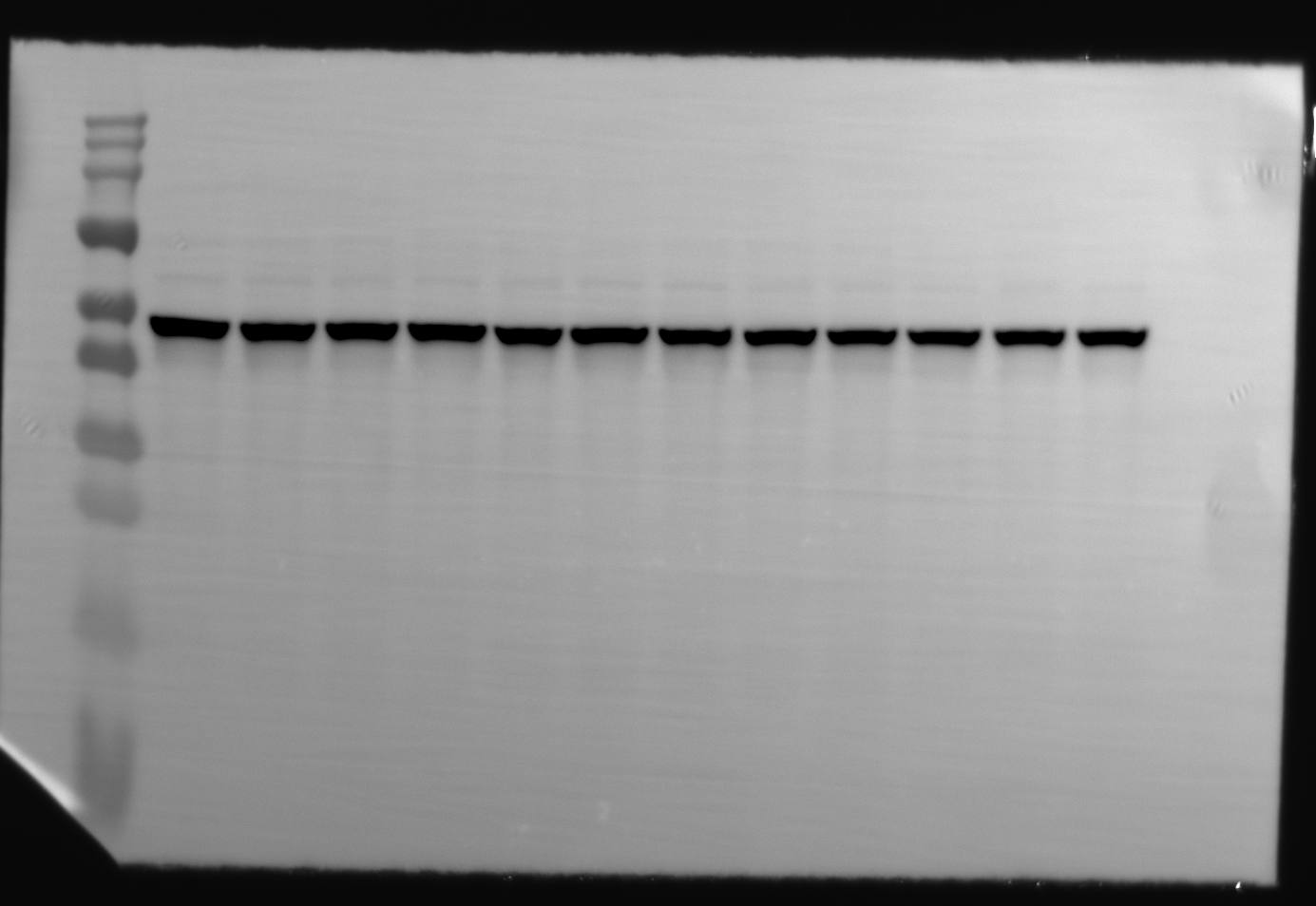

Supplement: Supplementary file 1 [file DataSheet1.ZIP › WB/Figure2/β-actin/β-actin 2.tif]

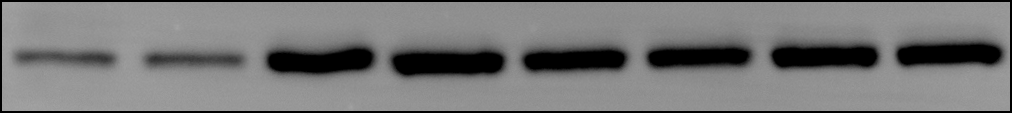

Supplement: Supplementary file 1 [file DataSheet1.ZIP › WB/Figure4/ASC/ASC 1-1.tif]

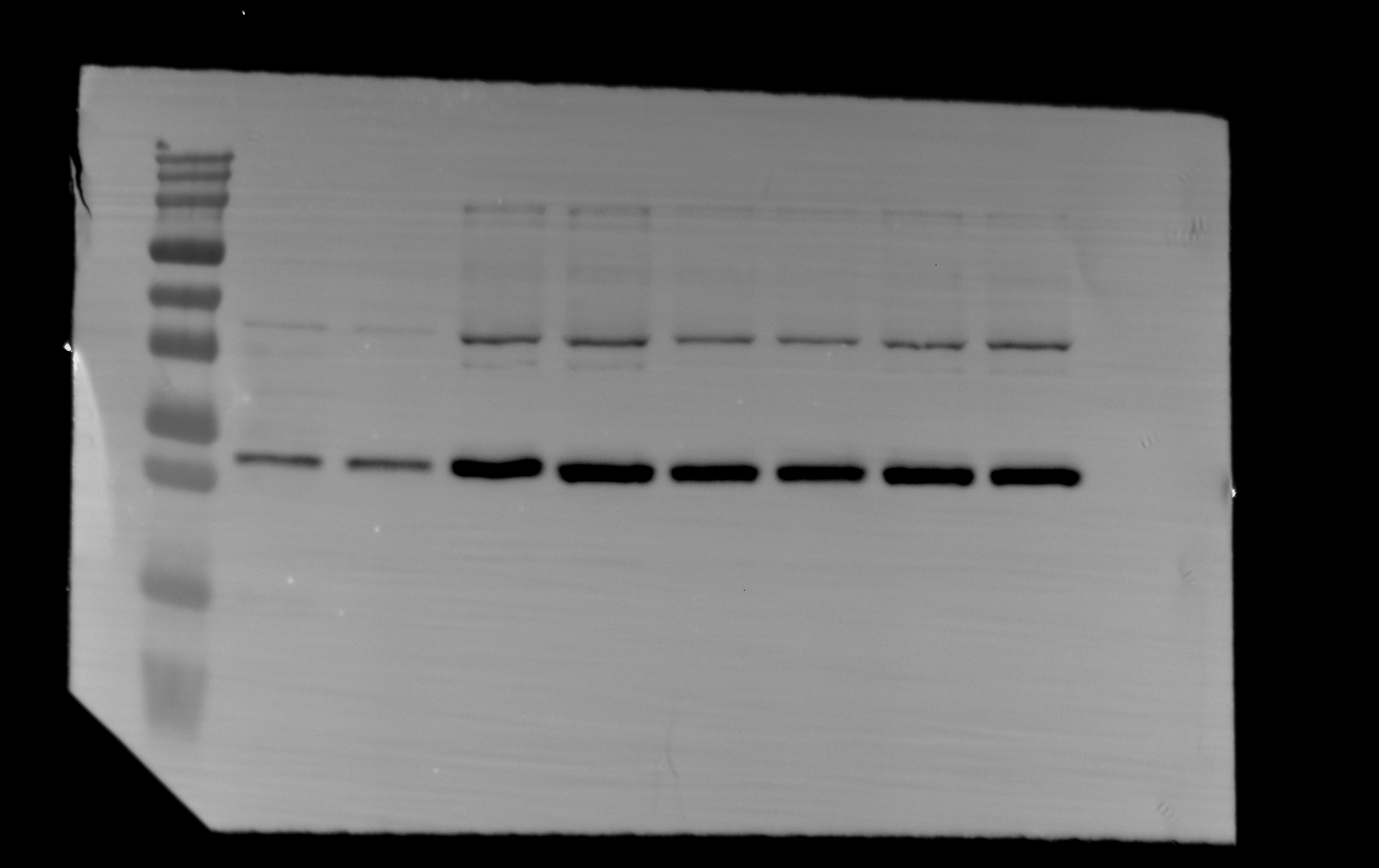

Supplement: Supplementary file 1 [file DataSheet1.ZIP › WB/Figure4/ASC/ASC 1.tif]

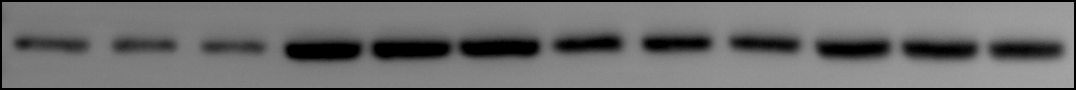

Supplement: Supplementary file 1 [file DataSheet1.ZIP › WB/Figure4/ASC/ASC 2-1.tif]

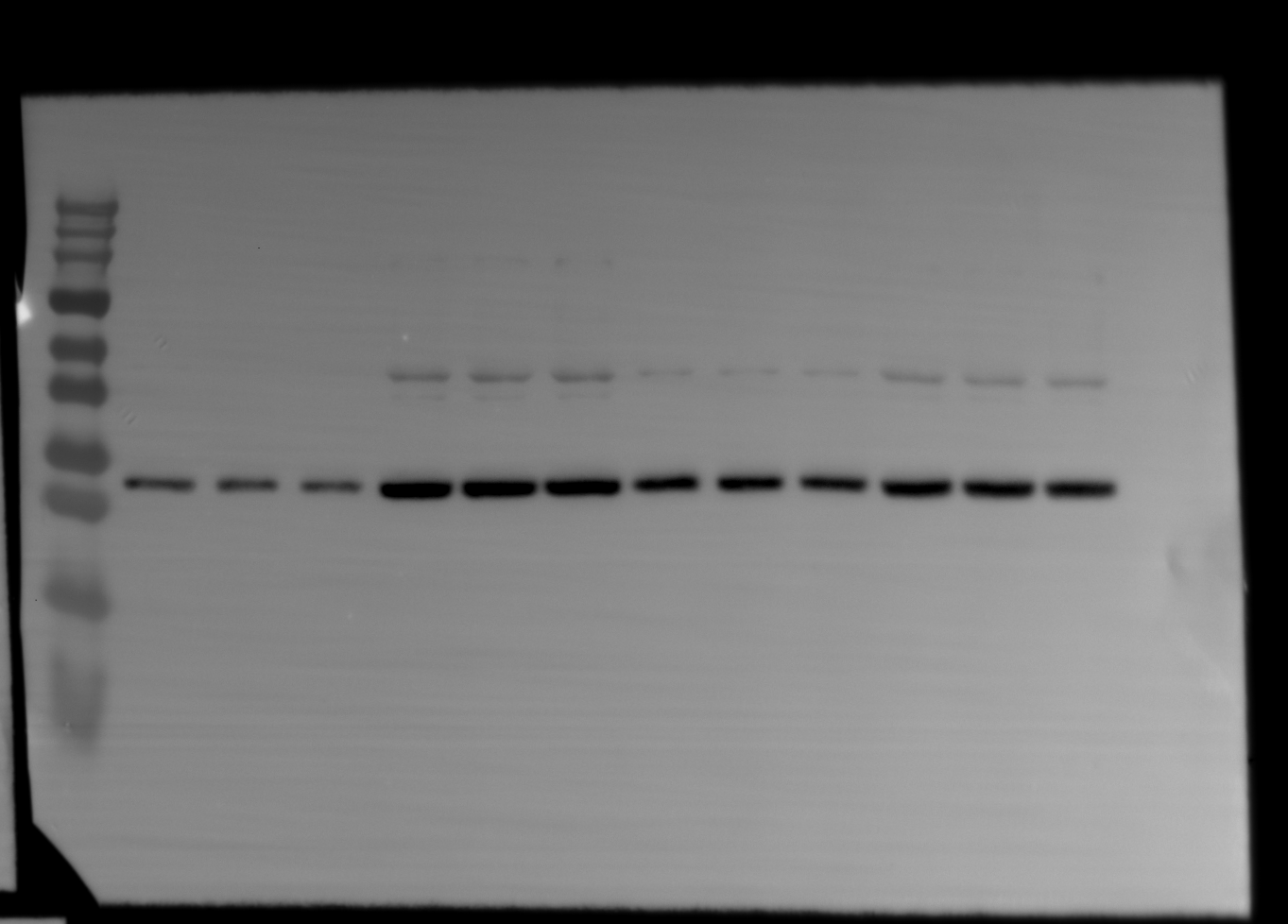

Supplement: Supplementary file 1 [file DataSheet1.ZIP › WB/Figure4/ASC/ASC 2.tif]

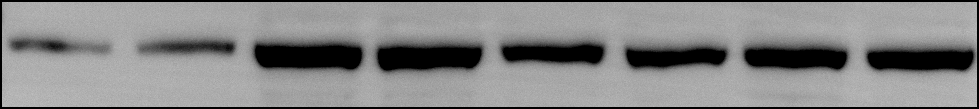

Supplement: Supplementary file 1 [file DataSheet1.ZIP › WB/Figure4/Caspase1/Caspase1-1-1.tif]

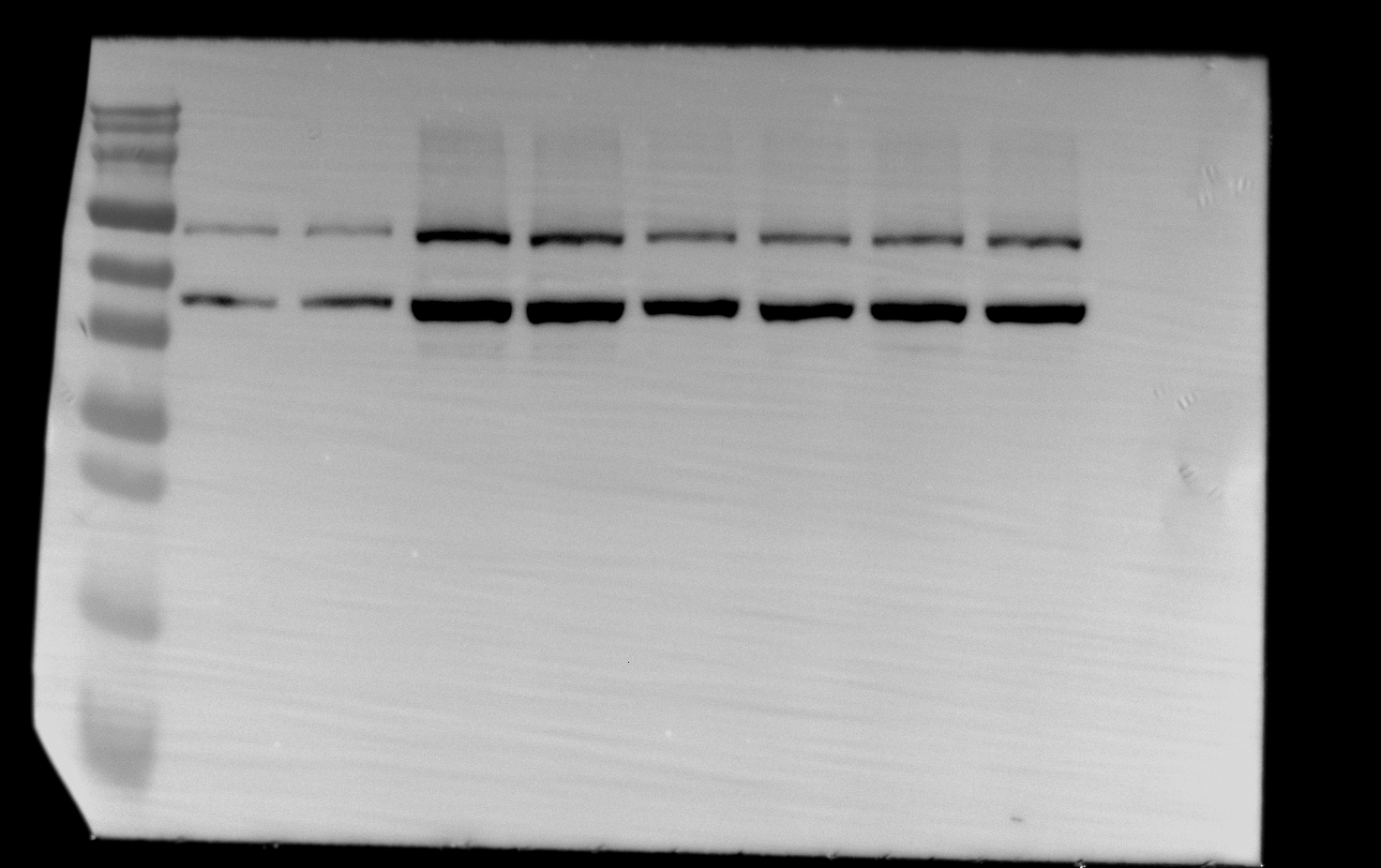

Supplement: Supplementary file 1 [file DataSheet1.ZIP › WB/Figure4/Caspase1/Caspase1-1.tif]

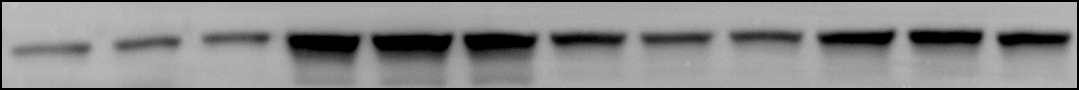

Supplement: Supplementary file 1 [file DataSheet1.ZIP › WB/Figure4/Caspase1/Caspase1-2-1.tif]

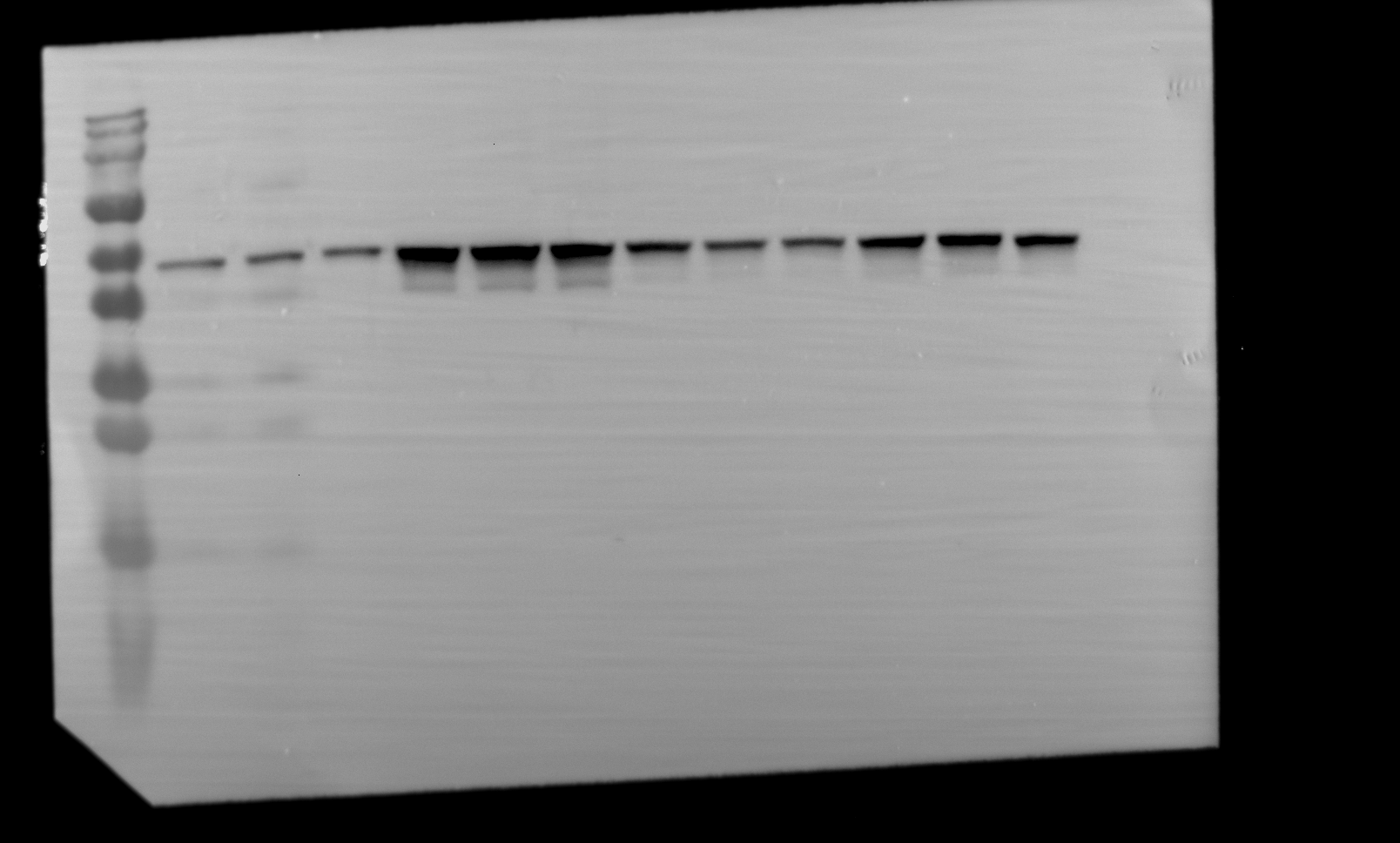

Supplement: Supplementary file 1 [file DataSheet1.ZIP › WB/Figure4/Caspase1/Caspase1-2.tif]

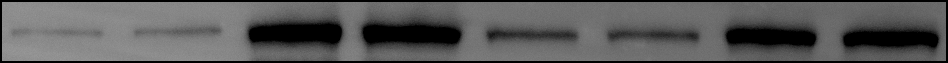

Supplement: Supplementary file 1 [file DataSheet1.ZIP › WB/Figure4/NLRP3/NLRP3 1-1.tif]

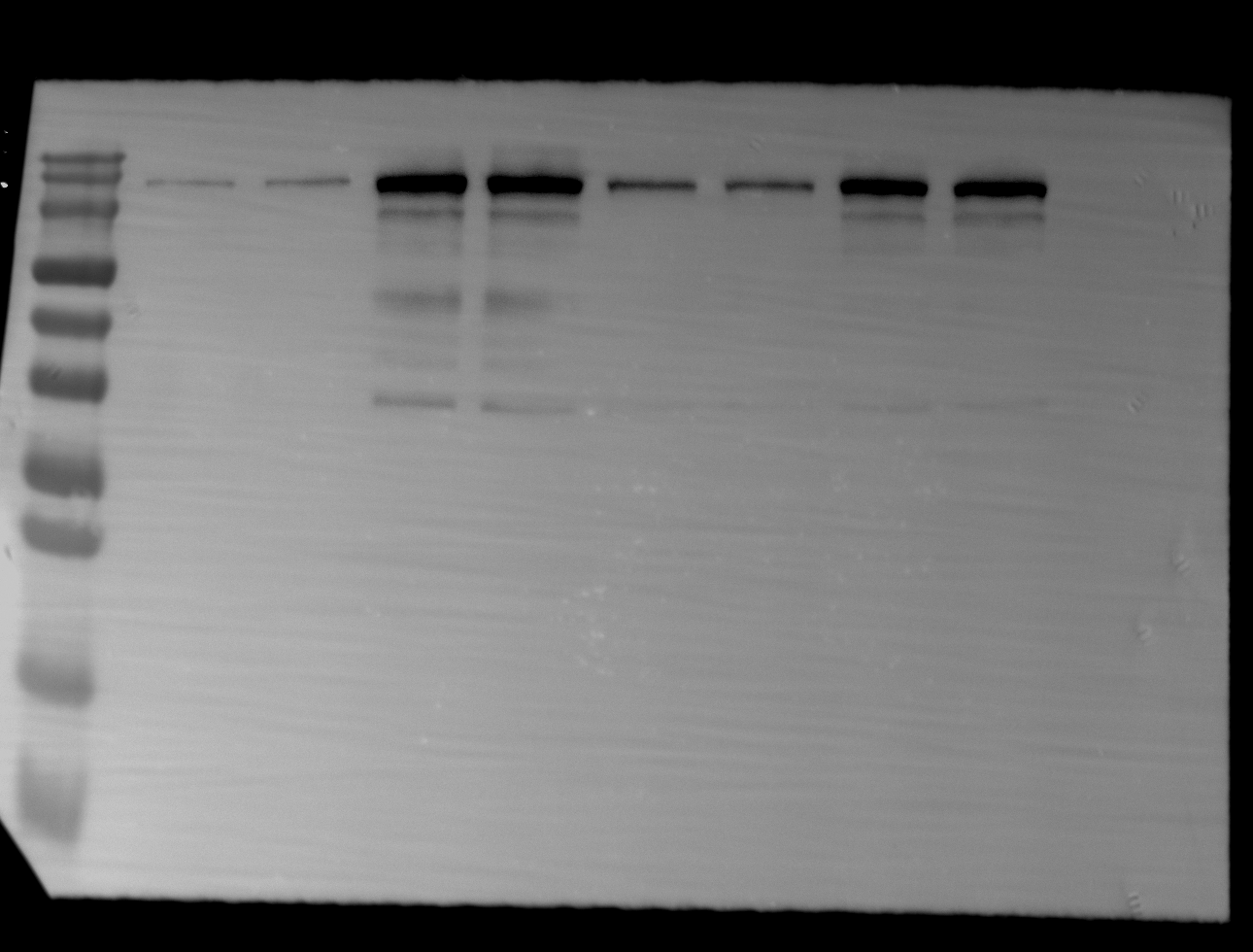

Supplement: Supplementary file 1 [file DataSheet1.ZIP › WB/Figure4/NLRP3/NLRP3 1.tif]

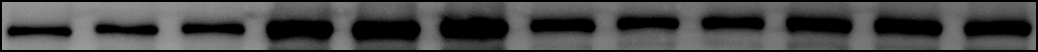

Supplement: Supplementary file 1 [file DataSheet1.ZIP › WB/Figure4/NLRP3/NLRP3 2-1.tif]

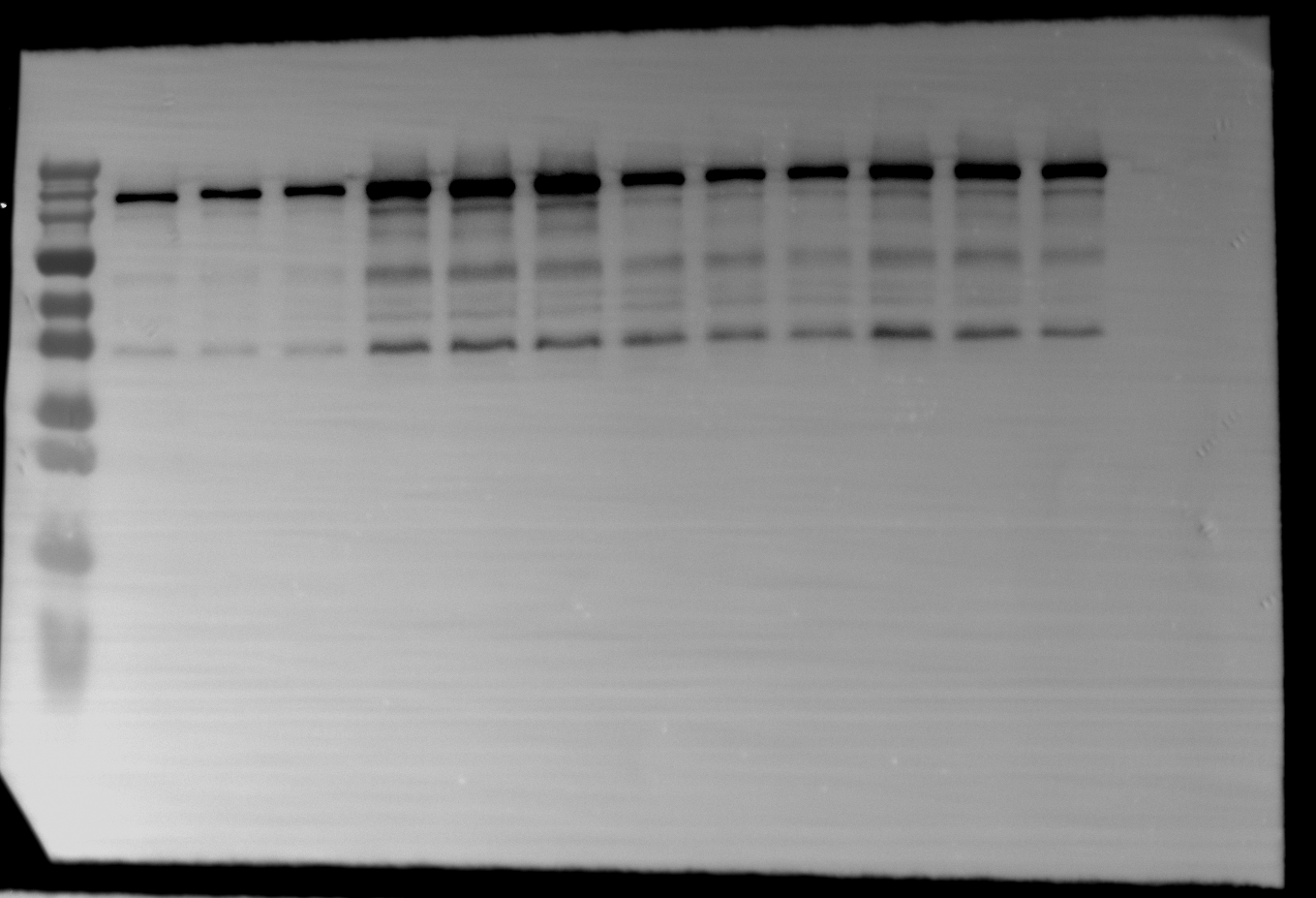

Supplement: Supplementary file 1 [file DataSheet1.ZIP › WB/Figure4/NLRP3/NLRP3 2.tif]

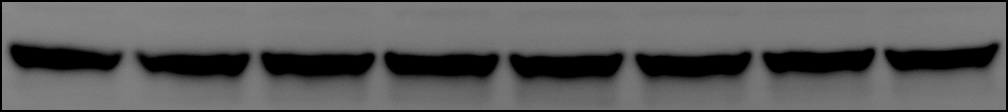

Supplement: Supplementary file 1 [file DataSheet1.ZIP › WB/Figure4/β-actin/β-actin 3-1.tif]

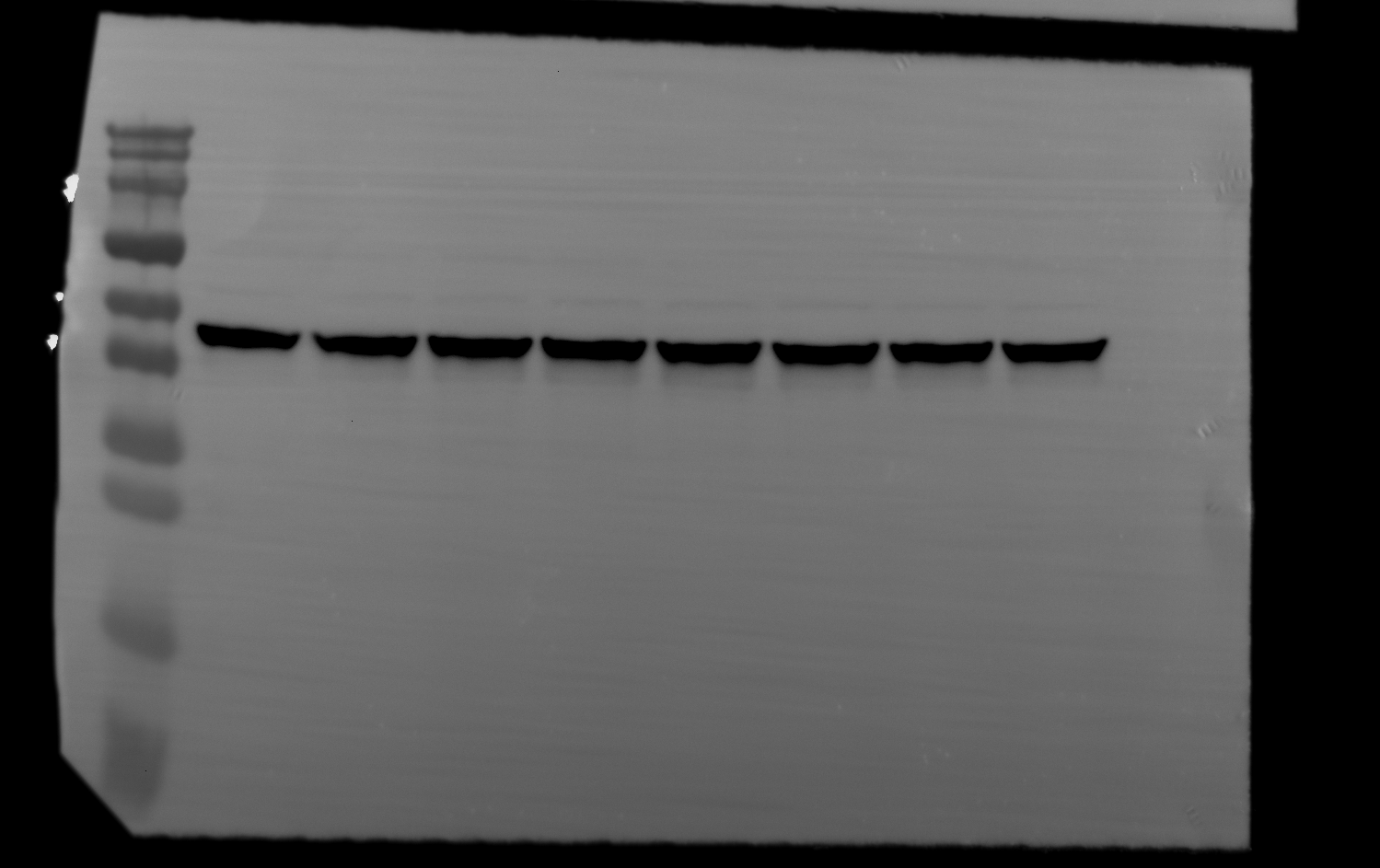

Supplement: Supplementary file 1 [file DataSheet1.ZIP › WB/Figure4/β-actin/β-actin 3.tif]

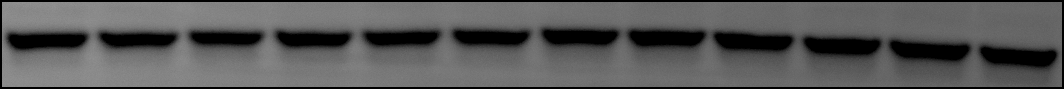

Supplement: Supplementary file 1 [file DataSheet1.ZIP › WB/Figure4/β-actin/β-actin 4-1.tif]

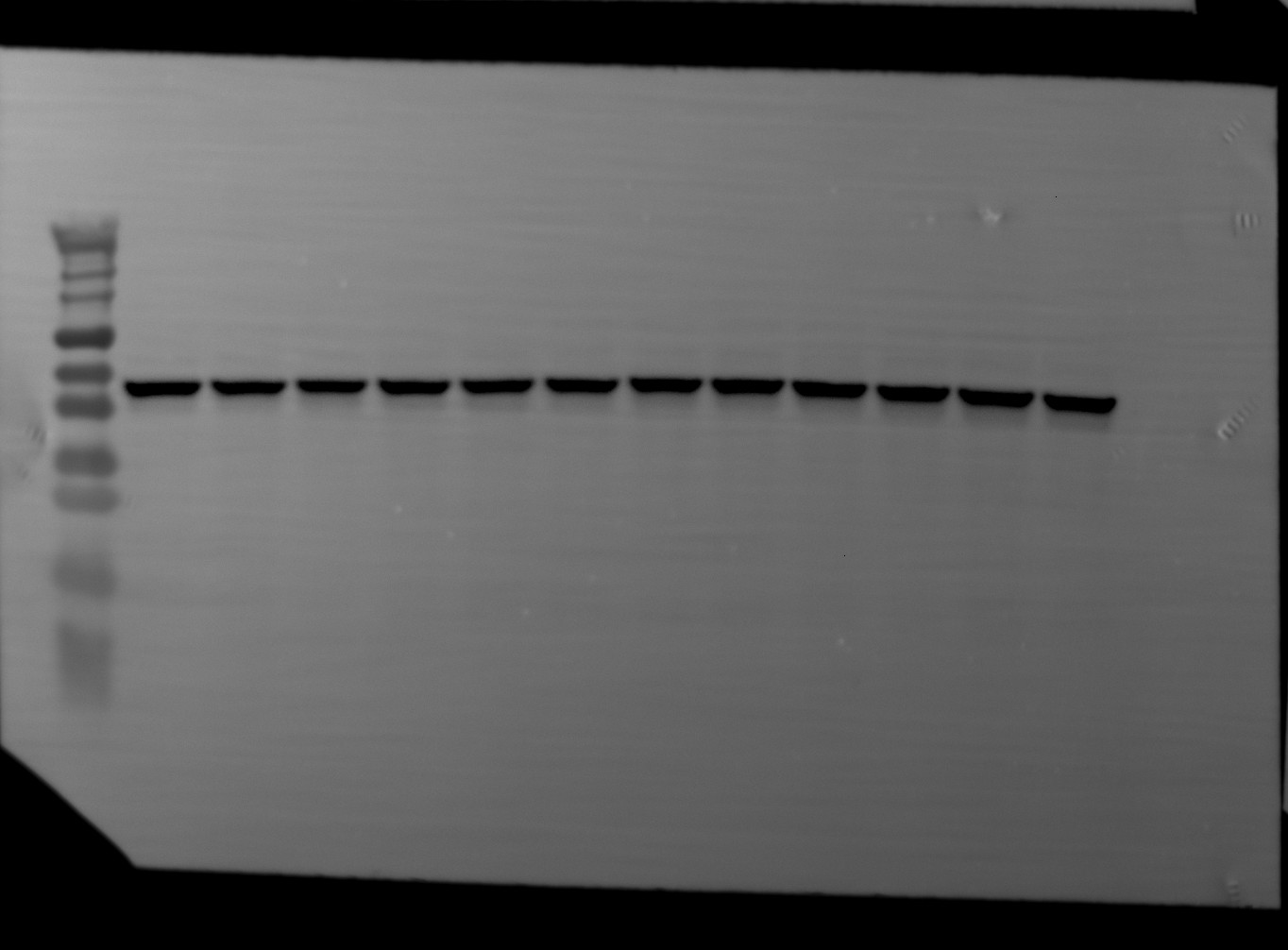

Supplement: Supplementary file 1 [file DataSheet1.ZIP › WB/Figure4/β-actin/β-actin 4.tif]

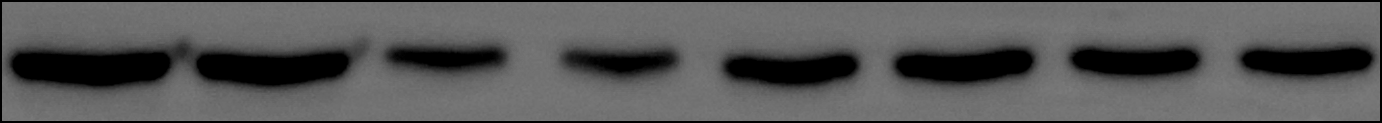

Supplement: Supplementary file 1 [file DataSheet1.ZIP › WB/Figure5/Claudin-1/Claudin-1-1-1一.tif]

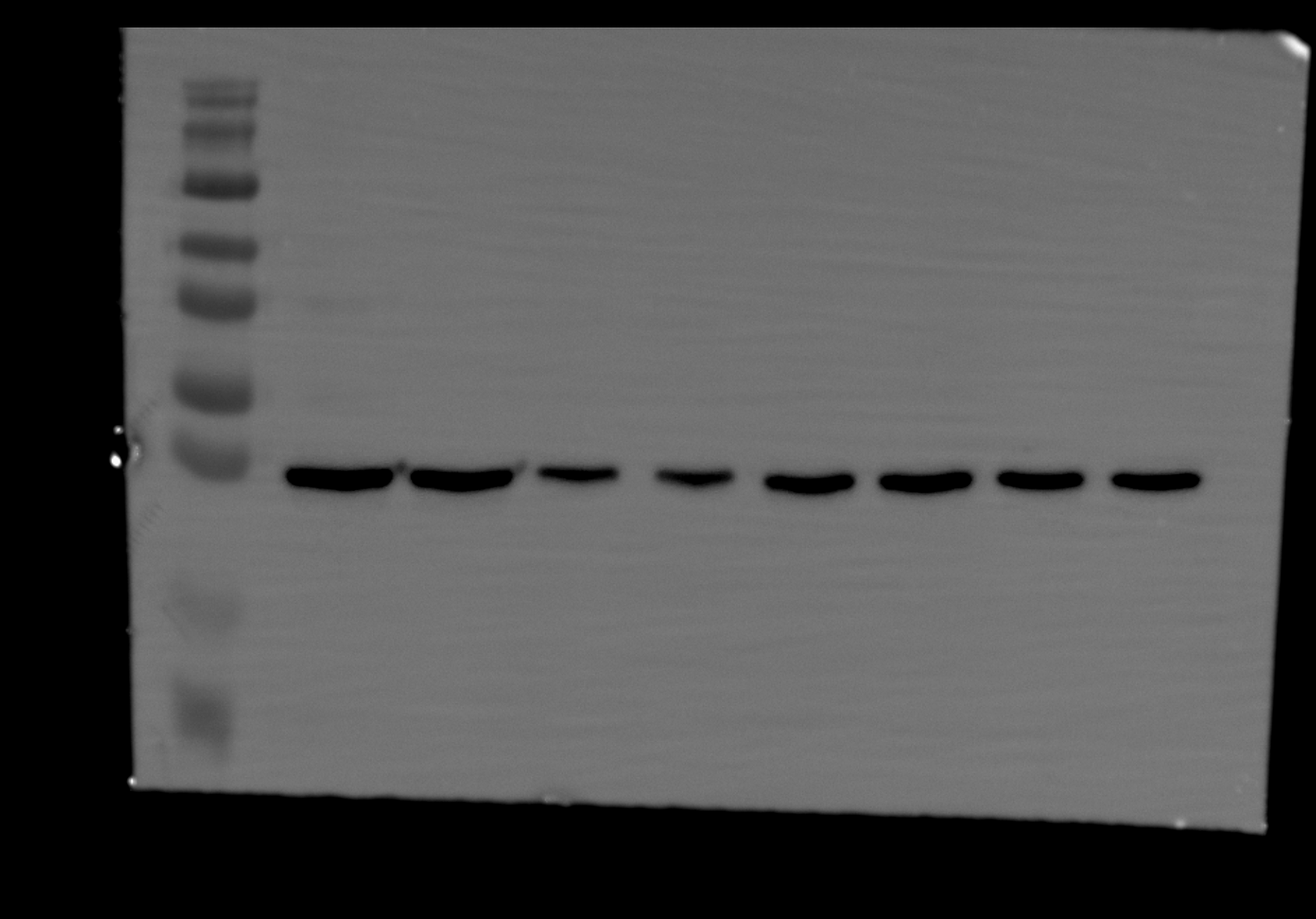

Supplement: Supplementary file 1 [file DataSheet1.ZIP › WB/Figure5/Claudin-1/Claudin-1-1.tiff]

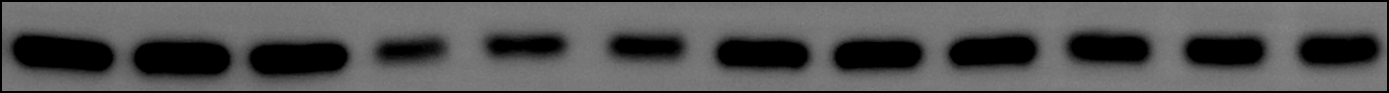

Supplement: Supplementary file 1 [file DataSheet1.ZIP › WB/Figure5/Claudin-1/Claudin-1-2-1一.tif]

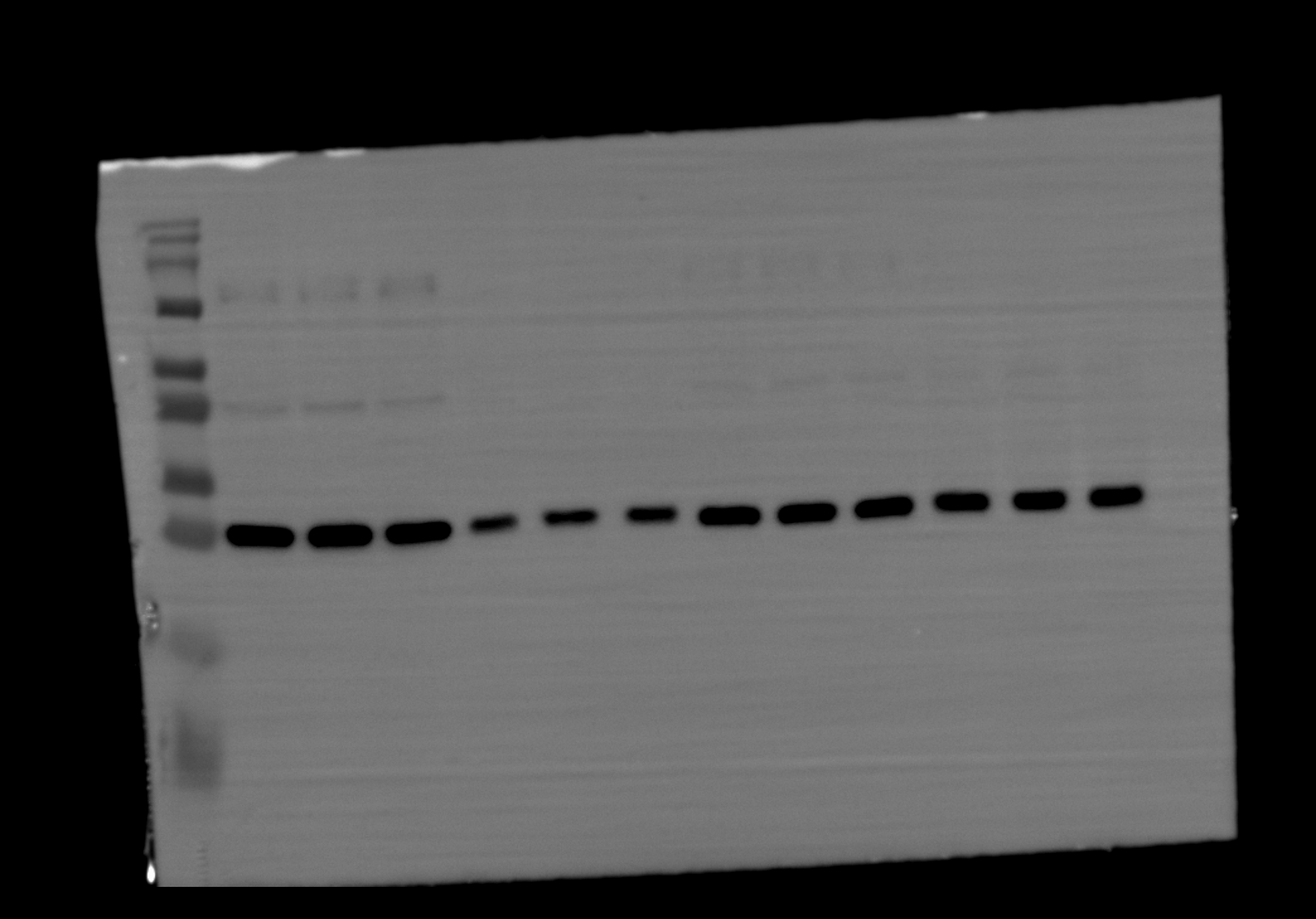

Supplement: Supplementary file 1 [file DataSheet1.ZIP › WB/Figure5/Claudin-1/Claudin-1-2.tiff]

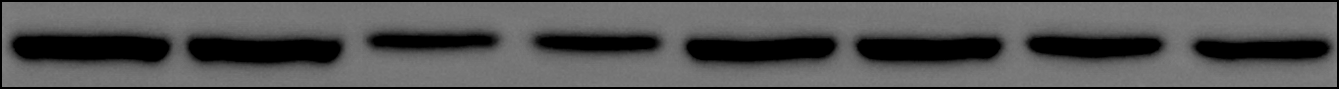

Supplement: Supplementary file 1 [file DataSheet1.ZIP › WB/Figure5/Occludin/Occludin-1-1.tif]

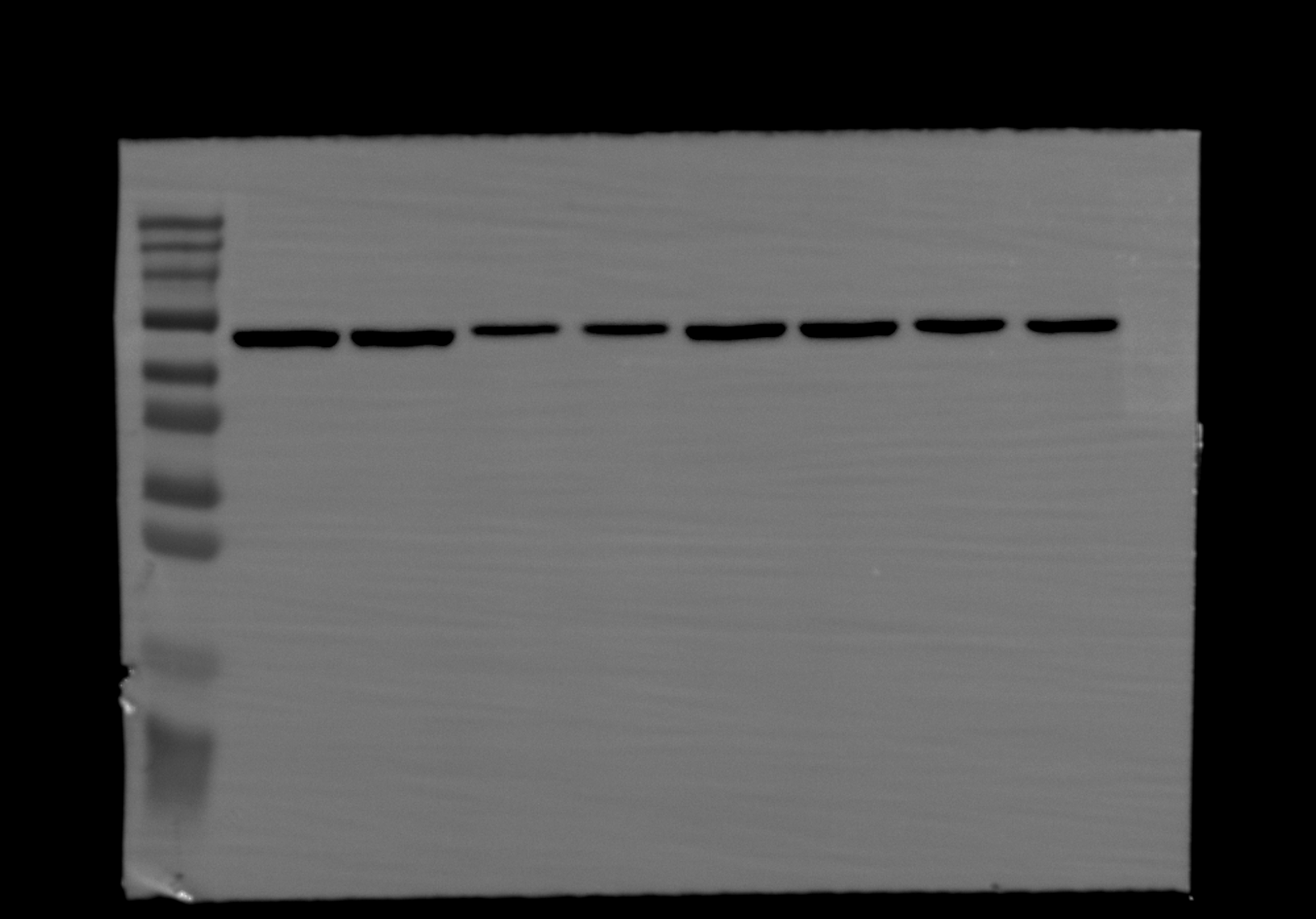

Supplement: Supplementary file 1 [file DataSheet1.ZIP › WB/Figure5/Occludin/Occludin-1.tiff]

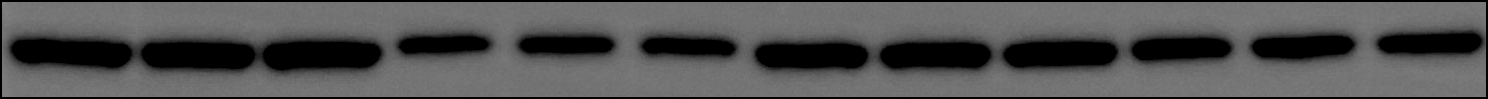

Supplement: Supplementary file 1 [file DataSheet1.ZIP › WB/Figure5/Occludin/Occludin-2.tif]

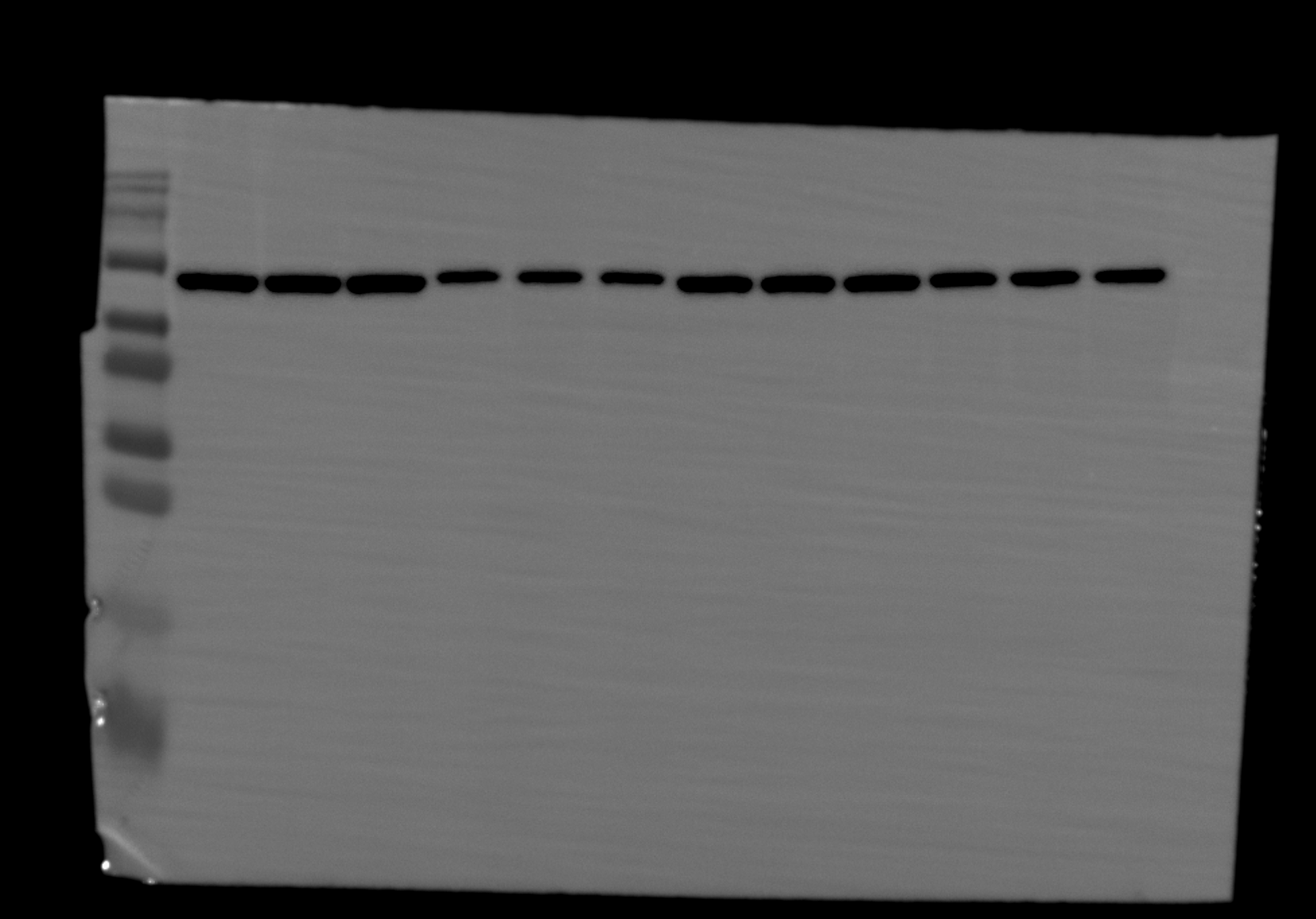

Supplement: Supplementary file 1 [file DataSheet1.ZIP › WB/Figure5/Occludin/Occludin-2.tiff]

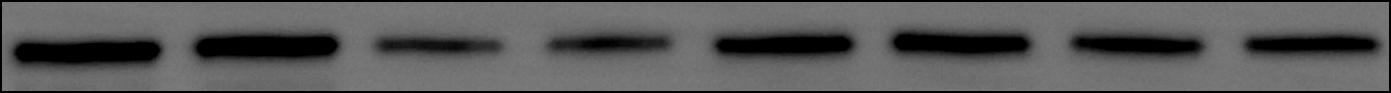

Supplement: Supplementary file 1 [file DataSheet1.ZIP › WB/Figure5/ZO-1/ZO-1-1-1.tif]

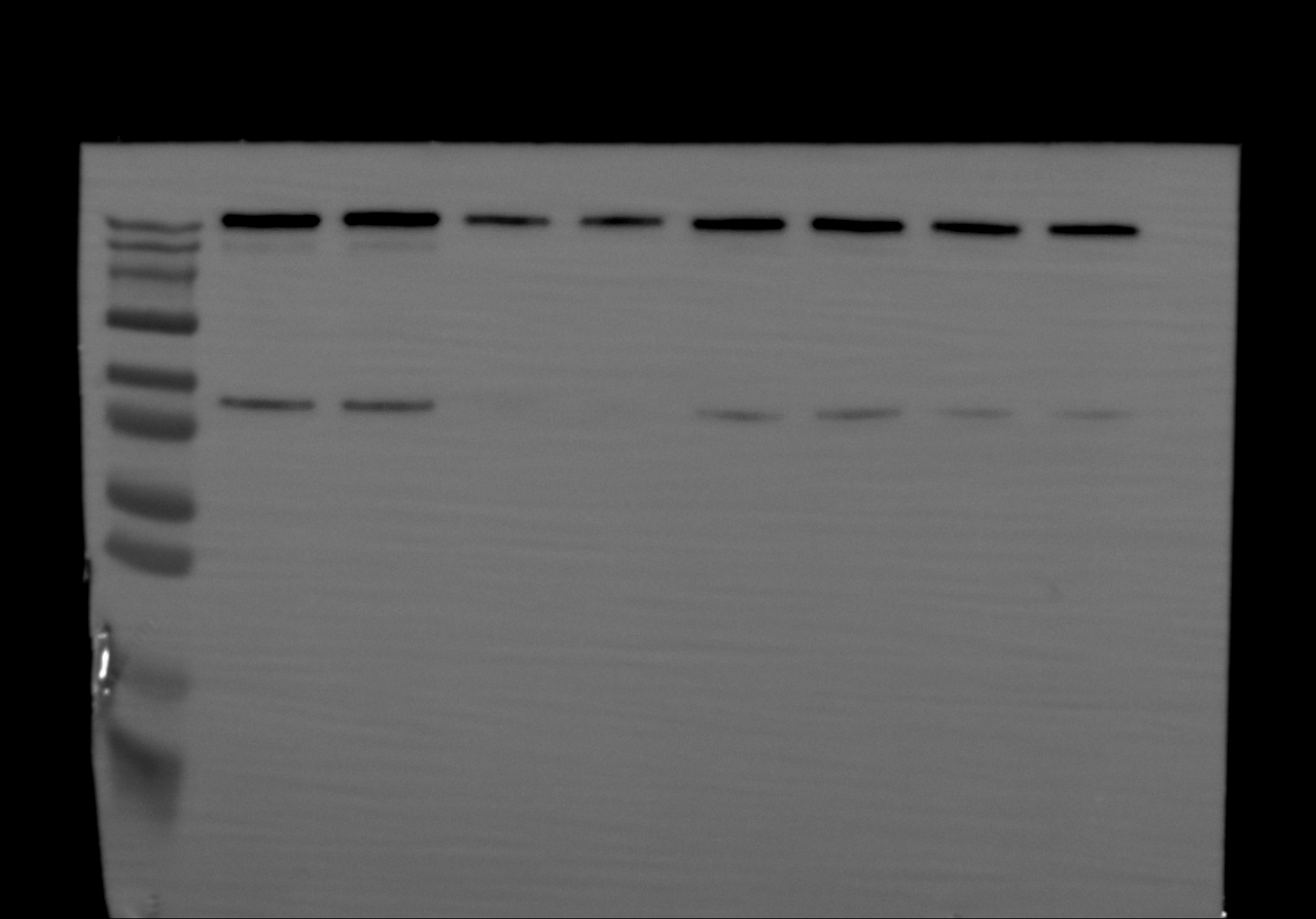

Supplement: Supplementary file 1 [file DataSheet1.ZIP › WB/Figure5/ZO-1/ZO-1-1.tiff]

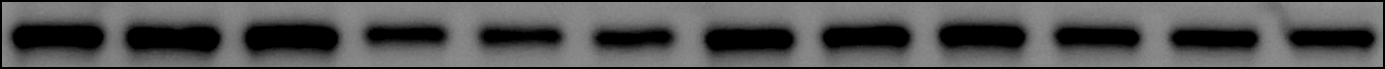

Supplement: Supplementary file 1 [file DataSheet1.ZIP › WB/Figure5/ZO-1/ZO-1-2-1.tif]

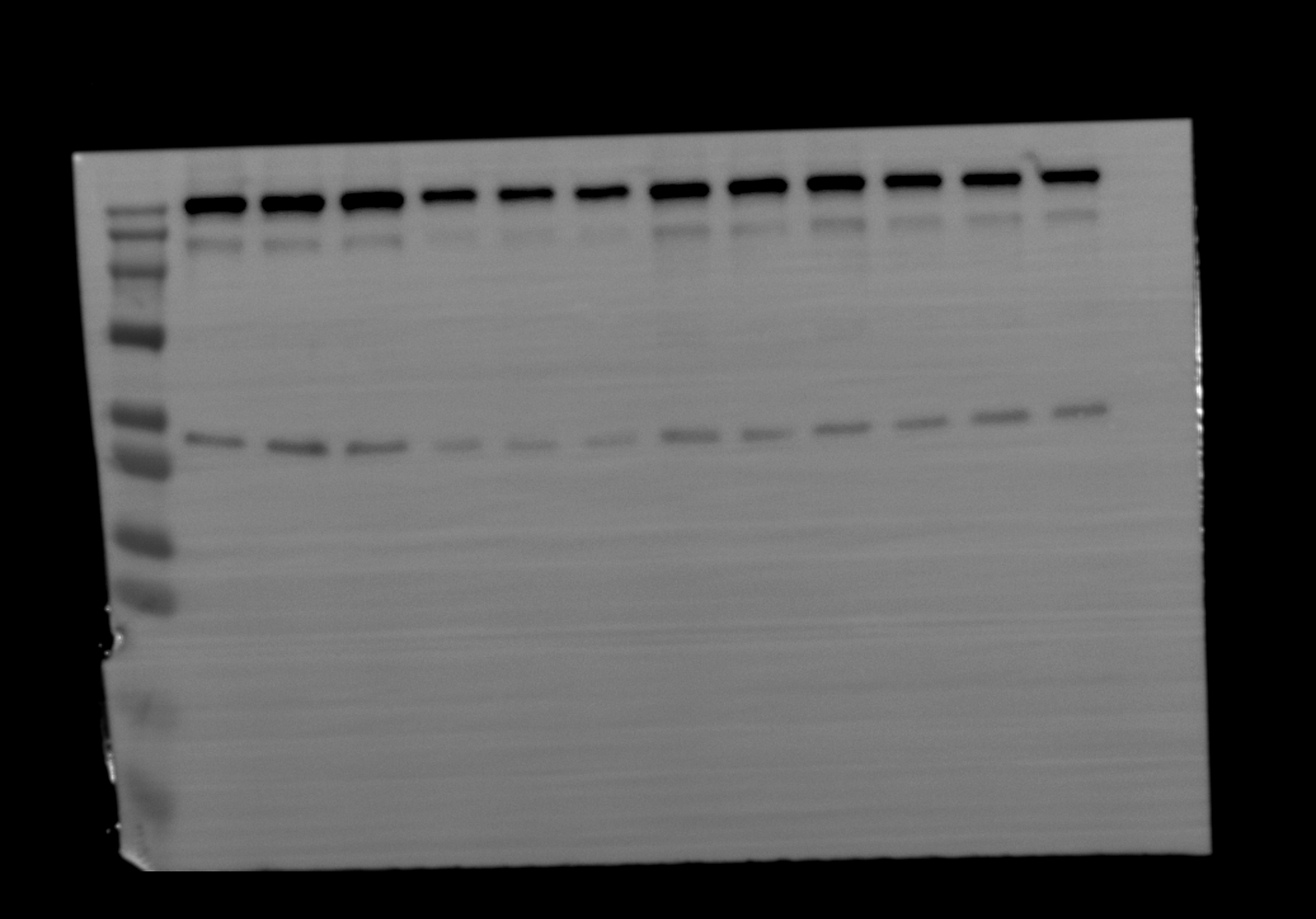

Supplement: Supplementary file 1 [file DataSheet1.ZIP › WB/Figure5/ZO-1/ZO-1-2.tiff]

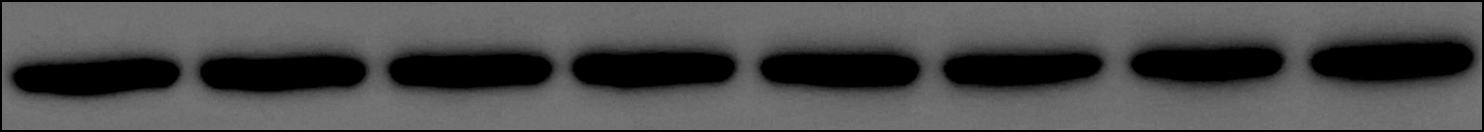

Supplement: Supplementary file 1 [file DataSheet1.ZIP › WB/Figure5/β-actin/β-actin 5-1.tif]

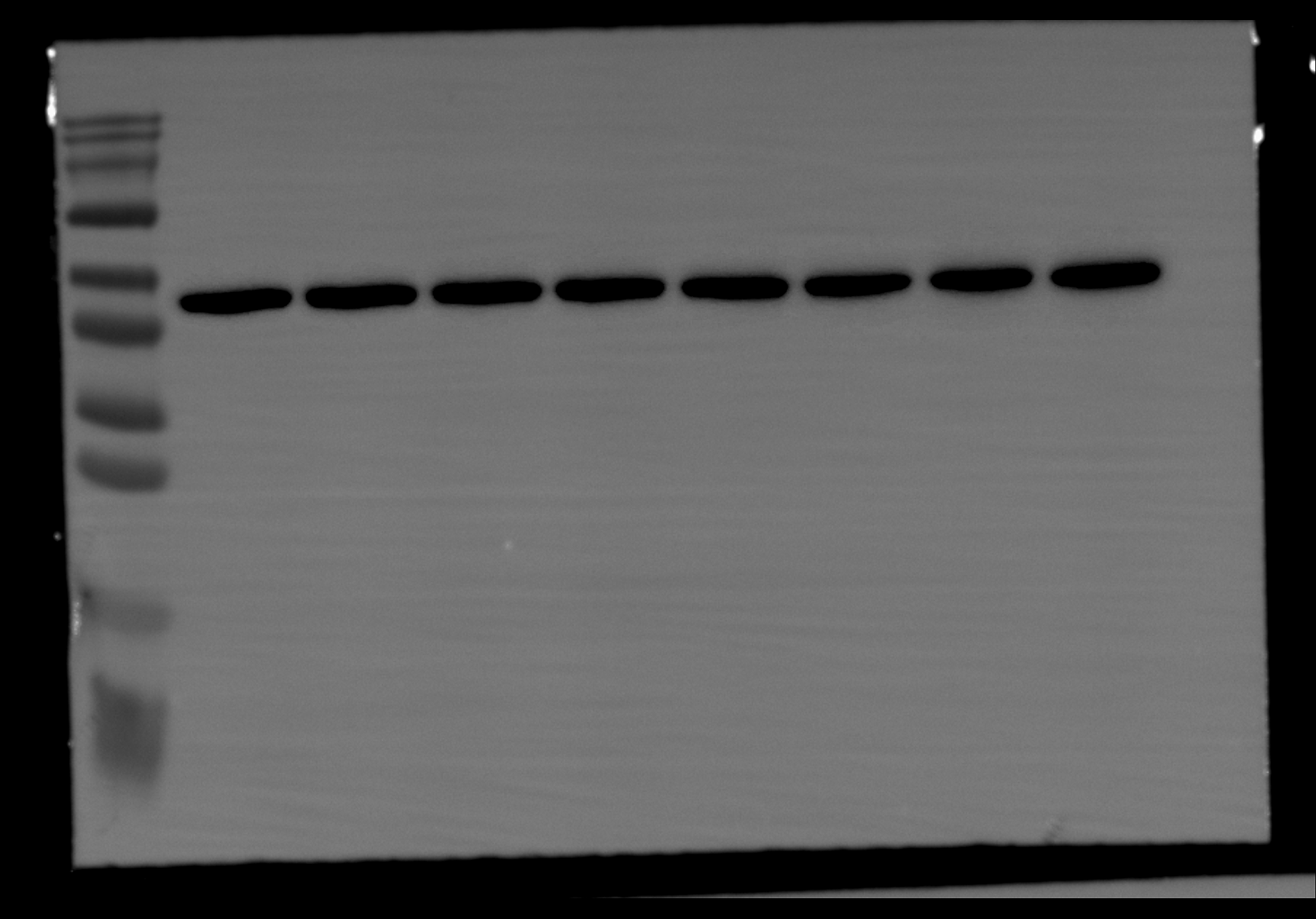

Supplement: Supplementary file 1 [file DataSheet1.ZIP › WB/Figure5/β-actin/β-actin 5.tiff]

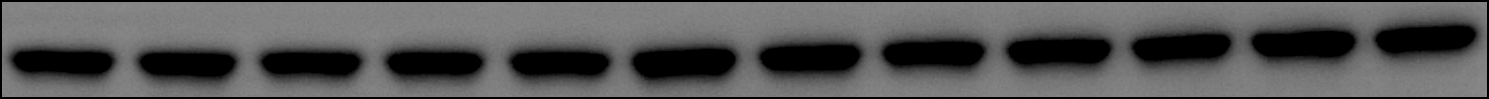

Supplement: Supplementary file 1 [file DataSheet1.ZIP › WB/Figure5/β-actin/β-actin 6-1.tif]

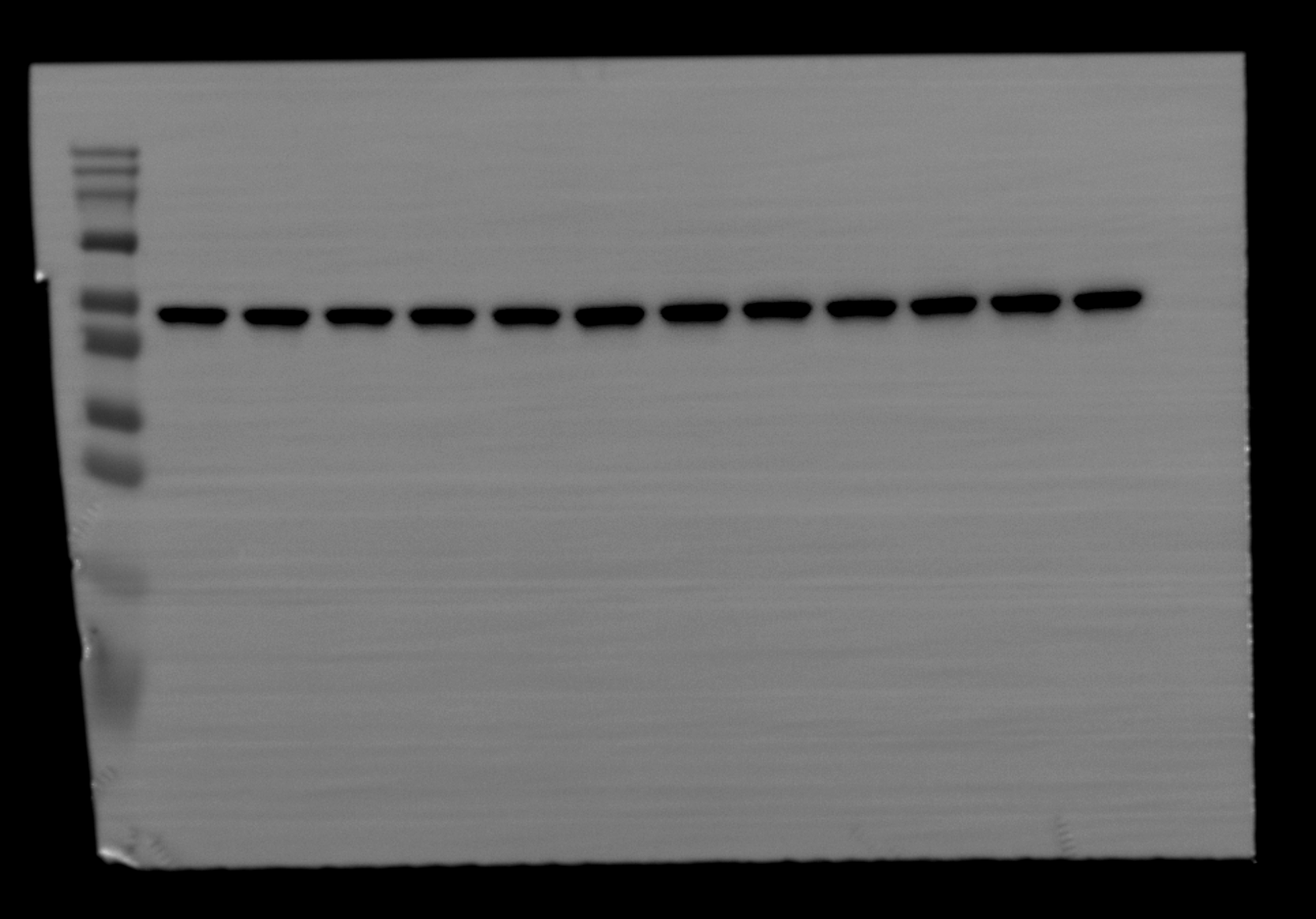

Supplement: Supplementary file 1 [file DataSheet1.ZIP › WB/Figure5/β-actin/β-actin 6.tiff]
